# Differences in trajectories of quality of life according to type of dementia: 6-year longitudinal findings from the IDEAL programme

**DOI:** 10.1186/s12916-024-03492-y

**Published:** 2024-06-24

**Authors:** Anthony Martyr, Laura D. Gamble, Anna Hunt, Catherine Quinn, Robin G. Morris, Catherine Henderson, Louise Allan, Carol Opdebeeck, Catherine Charlwood, Roy W. Jones, Claire Pentecost, Michael D. Kopelman, Jeanette M. Thom, Fiona E. Matthews, Linda Clare

**Affiliations:** 1 Centre for Research in Ageing and Cognitive Health, University of Exeter Medical School, Exeter, UK; 2 Population Health Sciences Institute, Newcastle University, Biomedical Research Building, Campus for Ageing and Vitality, Newcastle Upon Tyne, UK; 3 Centre for Applied Dementia Studies, Bradford University, Bradford, UK; 4 Wolfson Centre for Applied Health Research, Bradford, UK; 5 Department of Psychology, Institute of Psychiatry, Psychology and Neuroscience, King’s College London, London, UK; 6 Care Policy and Evaluation Centre, London School of Economics and Political Science, London, UK; 7 NIHR Applied Research Collaboration South-West Peninsula, Exeter, UK; 8 Department of Psychology, Manchester Metropolitan University, Manchester, UK; 9 Research Institute for the Care of Older People (RICE), Bath, UK; 10 Department of Psychological Medicine, Institute of Psychiatry, Psychology and Neuroscience, King’s College London, London, UK; 11 School of Health Sciences, The University of Sydney, Sydney, Australia; 12 Institute for Clinical and Applied Health Research, Hull York Medical School, University of Hull, Hull, UK

**Keywords:** Living well, Well-being, Satisfaction with life, Lewy body dementia, Carer, Caregiver, Longitudinal

## Abstract

**Background:**

People with different types of dementia may have distinct symptoms and experiences that affect their quality of life. This study investigated whether quality of life varied across types of dementia and over time.

**Methods:**

The participants were 1555 people with mild-to-moderate dementia and 1327 carers from the IDEAL longitudinal cohort study, recruited from clinical services. As many as possible were followed for up to 6 years. Diagnoses included were Alzheimer’s disease, vascular dementia, mixed Alzheimer’s and vascular dementia, Parkinson’s disease dementia, dementia with Lewy bodies, and frontotemporal dementia. Self- and informant-rated versions of the Quality of Life in Alzheimer’s Disease scale were used. A joint model, incorporating a mixed effects model with random effects and a survival model to account for dropout, was used to examine whether quality of life varied by dementia type at the time of diagnosis and how trajectories changed over time.

**Results:**

The strongest associations between dementia type and quality of life were seen around the time of diagnosis. For both self-ratings and informant ratings, people with Parkinson’s disease dementia or dementia with Lewy bodies had lower quality of life scores. Over time there was little change in self-rated scores across all dementia types (− 0.15 points per year). Informant-rated scores declined over time (− 1.63 points per year), with the greatest decline seen in ratings by informants for people with dementia with Lewy bodies (− 2.18 points per year).

**Conclusions:**

Self-rated quality of life scores were relatively stable over time whilst informant ratings showed a steeper decline. People with Parkinson’s disease dementia or dementia with Lewy bodies report particularly low levels of quality of life, indicating the importance of greater attention to the needs of these groups.

**Supplementary Information:**

The online version contains supplementary material available at 10.1186/s12916-024-03492-y.

## Background

Dementia is a global public health priority. There are 55 million people currently living with dementia worldwide, a number estimated to increase to 139 million by 2050 [1]. Dementia comprises over 100 conditions [2], with the most frequent diagnoses being Alzheimer’s disease (AD), vascular dementia (VaD), mixed Alzheimer’s and vascular dementia (mixed dementia), Parkinson’s disease dementia (PDD), dementia with Lewy bodies (DLB), and frontotemporal dementia (FTD) [3]. PDD, DLB, and FTD remain relatively less common, each accounting for around 2–3% of all dementia diagnoses [3, 4]. However, the prevalence of DLB may be higher due to misdiagnosis rate of around 20%, as it is often mistaken for AD [5]. Each type of dementia has a different aetiology and trajectory with concomitant impacts on health care needs and post-diagnostic support [6]. To develop, offer, and optimise tailored care and support services, it is important to identify factors that affect quality of life [7, 8]. People in some diagnostic groups may be at higher risk of poor quality of life due to their specific range of symptoms.

Factors that relate to quality of life in dementia have been comprehensively reviewed [9]. Numerous factors influence self-rated and informant-rated quality of life, but these effects are generally small in degree. Most previous studies have included small sample sizes, especially those studies that cover the less common types of dementia [10–12]. Studies considering differences among diagnostic groups have focused either on comparing a few types of dementia, primarily AD with one or two other types [10, 13–18], or on comparing AD with a single heterogeneous group that comprises various other dementia diagnoses [12, 19–21]. This makes it difficult to understand how quality of life differs among people with diagnoses other than AD, although a few studies have investigated quality of life in a single non-AD dementia such as PDD [22] or DLB [23, 24]. Using baseline data from our large, longitudinal cohort study—Improving the experience of Dementia and Enhancing Active Life (IDEAL) [25, 26]—people with AD had higher quality of life scores than all other diagnostic groups, and people with PDD or DLB had the lowest scores [27].

Changes in cognitive and functional abilities as dementia progresses [28] might be reflected in changed perceptions of quality of life, but relatively few studies have investigated how quality of life changes over time for people with dementia [9]. To our knowledge, the only study to have explored whether change over time in quality of life differs among diagnostic groups is our earlier study also from IDEAL [7]. This study found that people with PDD/DLB had lower quality of life scores over 2 years. However, dementia type was investigated as one element in a wide range of factors that might influence self-rated quality of life. In addition, people with PDD or DLB were treated as a single group, which could limit the applicability of those findings, especially given that as we have already noted people with PDD or DLB score lower on quality of life than people with other dementia diagnoses [27]. A more focused investigation of the differences between diagnostic groups is needed.

The present study extends our two previous studies [7, 27] in several ways. The present study extends the possible number of years of follow-up from 2 to 6 years, includes additional people with less common dementia diagnoses, investigates changes in informant-rated as well as self-rated quality of life, and estimates scores on these measures from the time of diagnosis. The aim of the present study is therefore to compare changes in self-rated and informant-rated quality of life over 6 years in six diagnostic groups: AD, VaD, mixed dementia, PDD, DLB, and FTD.

## Methods

### Design

The IDEAL programme established and followed a longitudinal cohort of people with dementia and their carers in Britain [25, 26]. IDEAL was a longitudinal cohort study that recruited participants from clinical services. Data were collected between 2014 and 2021. This paper presents longitudinal data using version 7 of the datasets [29]. Over two recruitment waves, 1749 people with dementia together with 1460 carers, mostly spouses or partners [30], took part in the study. Assessments were conducted over six timepoints: time 1 (2014 to 2016), time 2 (2015 to 2017), time 3 (2016 to 2018), time 4 (2018 to 2020), time 5 (2019 to 2020), and time 6 (2021). Data collection at times 4 to 6 was disrupted by the COVID-19 pandemic [31]. People with dementia were recruited through 34 National Health Service research networks across England, Scotland, and Wales. To meet inclusion criteria for entry to the study, participants had to have a clinical diagnosis of any type of dementia as judged by clinicians at recruitment sites, a score of 15 or above on the Mini-Mental State Examination (MMSE) [32] indicating mild-to-moderate dementia, and the ability to communicate verbally in English. Exclusion criteria at entry were co-morbid terminal illness and inability to provide informed consent. Carers of people with dementia took part in IDEAL if the person with dementia they cared for also took part. People with dementia once recruited could nominate a carer to participate alongside them. A carer was defined as the primary person who provides practical or emotional unpaid support, usually a family member [33]. There were no specific inclusion criteria for carers other than being willing to take part. Carers provided informant ratings about the person with dementia and information about their own caring experiences. For the present study, informant-rated quality of life scores provided by carers were used alongside self-ratings provided by people with dementia. Full criteria for exclusion and consent are provided in the protocol [25]. IDEAL was approved by the Wales Research Ethics Committee 5 (reference 13/WA/0405) and the Ethics Committee of the School of Psychology, Bangor University (reference 2014–11684). IDEAL-2 was approved by Wales Research Ethics Committee 5 (reference 18/WA/0111) and Scotland A Research Ethics Committee (reference 18/SS/0037). The studies were registered with UKCRN, registration numbers 16593 (IDEAL) and 37955 (IDEAL-2).

### Quality of life

Quality of life was assessed using the Quality of Life in AD (QoL-AD) [34] scale. This measure comprises 13 questions assessing perceptions of various aspects of everyday life. Responses to each question are ‘poor’, ‘fair’, ‘good’, or ‘excellent’. Scores range between 13 and 52 with higher scores indicating better quality of life. This measure is widely used [9] and the present study uses both self- and informant-rated versions.

### Diagnostic groups

Participants in IDEAL were diagnosed with one of seven types of dementia: AD, VaD, mixed dementia, FTD, PDD, DLB, or unspecified/other. Diagnosis was recorded by research staff from medical records. For the purposes of the present study, the unspecified/other group was excluded (*N* = 38) as this group comprised people with no specific type of dementia or with a very rare type of dementia. Time since diagnosis in years (to 2 decimal points) was calculated by subtracting the date of diagnosis from the date of each IDEAL interview. Those with missing date of diagnosis information were also excluded (*N* = 161).

### Participant characteristics

Characteristics used in the study comprised sex, age group at time of diagnosis (< 65, 65–69, 70–74, 75–79, 80 +), and kin relationship between the person with dementia and the carer taking part (spouse/partner or family/friend).

### Cognition

MMSE was used to measure level of cognitive function between time 1 to time 5. At time 6, the 5-min Montreal Cognitive Assessment [35] was used as this could be administered remotely as necessary due to COVID-19 restrictions; scores were converted to MMSE-equivalent scores at this timepoint [36]. Scores range between 0 and 30 for both measures with higher scores indicating better cognition.

### Statistical analysis

Descriptive statistics are reported for participant characteristics and for the longitudinal outcomes. Time since diagnosis was calculated for each participant for every timepoint at which they took part, using the date of interview and the date of diagnosis. For descriptive purposes only, time since diagnosis was grouped into bands: < 1 year, 1 to < 2 years, 2 to < 3 years, 3 to < 4 years, 4 to < 5 years, 5 to < 6 years, 6 to < 7 years, and 7 to < 8 years. Descriptive statistics are reported only up to the 7 to < 8 years post-diagnosis band due to low numbers in the subsequent years (for people with dementia, *n* = 35 for the 8 to < 9 year band, *n* = 16 for the 9 to < 10 year band, with 20 participants taking part 10 years or more post-diagnosis). However, all participants are included in the main analysis. A sensitivity analysis was conducted using only data up to 8 years post-diagnosis; see Additional file 1: Table S1. For the analyses, the exact time since diagnosis was used for each participant for each timepoint in which they took part. To assess longitudinal change in self-rated and informant-rated QoL-AD whilst accounting for bias due to non-random dropout, a joint longitudinal-survival model was conducted using the *JM* package in R [37].

First, a mixed effects model with random effects was specified for the longitudinal outcome, which estimates a latent intercept (year 0 of diagnosis) and slope (change per year following diagnosis). The key assumptions of the mixed effects model are linearity, homogeneity of variance, and normal distribution of the residuals. A standardised residual vs fitted values plot was inspected to test for linearity and homogeneity of variance, and the Q-Q plot was inspected to check the residuals for normality. Assumptions were met. Models incorporating a quadratic term, cubic term, or natural splines were also tested. The linear model had the best fit as determined by the Bayesian information criterion.

Second, a time-to-dropout Cox regression model was specified for the missingness process, which calculates a hazard ratio for an event (withdrawal/loss to follow-up) from the censored data. The Cox regression includes dementia type and the additional covariates specified for each model. The key assumption of this model is that the hazards are proportional over time (constant relative hazard). The proportional hazards assumption was tested using the *cox.zph* function in R and the assumption was met for each covariate. For the joint model, it is advisable to use a parametric but flexible model [37], so a proportional hazards model with a piecewise constant baseline risk function was specified.

In the joint model, the probability distributions from the two processes are combined and a set of random effects are assumed to account for the associations between the two outcomes. Full conditional independence is assumed. That is, that the random effects explain all interdependencies. The resulting estimated intercept and slope, which are controlled for dropout, are reported as the main findings. Full model results are reported in Additional file 1. Models for self-rated measures were adjusted for sex of the person with dementia (model 1), or sex and age group of the person with dementia (model 2), or sex, age group, and mean-centred MMSE score of the person with dementia (model 3). Models for informant-rated measures were additionally adjusted for kin relationship.

Reasons for dropout and non-participation at a given time are reported in Additional file 1: Table S2.

### Additional analyses

Whilst the primary focus of the present study is to investigate quality of life scores in people with dementia over 6 years, previously we have also examined satisfaction with life and psychological well-being both at baseline [27] and longitudinally over 2 years [7]. To explore these related but distinct constructs further, we conducted analysis that aligned with the quality of life modelling described above. These analyses used self-rated and informant-rated versions of the Satisfaction with Life Scale (SwLS) [38] and the World Health Organization-Five Well-Being Index (WHO-5) [39] to investigate satisfaction with life and psychological well-being, respectively. SwLS is a five-item scale designed to measure global judgements of satisfaction with life. Each question has seven possible responses that range from ‘strongly agree’ to ‘strongly disagree’. Scores range between 5 and 35 with higher scores indicating better satisfaction with life. WHO-5 is a five-item scale exploring psychological well-being. Each question has six responses ranging between ‘at no time’ to ‘all the time’. Scores range from 0 to 25 and are converted to a percentage scale with higher scores indicating better well-being.

## Results

Of the 1749 people with dementia and the 1460 carers that were recruited into the study, 161 people with dementia had a missing date of diagnosis and an additional 33 people with unspecified/other diagnoses were excluded. Consequently, the sample comprised 1555 people with dementia and 1327 carers; see Table 1 for sample characteristics. At the time of diagnosis, approximately half had a diagnosis of AD, just over half were male, and 40% were aged 80 or above; their carers were primarily their spouses/partners. Mean scores for self-rated and informant-rated QoL-AD, SwLS, and WHO-5 are shown in Additional file 1: Table S3. As shown in Table 1, the mean score for self-rated QoL-AD was 36.5 at baseline and remained relatively stable over time. The mean score for informant-rated QoL-AD was 34.0 at baseline and declined slightly to 30.7 at 7–8 years post-diagnosis. Self-rated QoL-AD mean scores were higher than the equivalent informant ratings.

**Table 1:** Descriptive statistics

	Years since diagnosis							
	< 1	1 to < 2	2 to < 3	3 to < 4	4 to < 5	5 to < 6	6 to < 7	7 to < 8
(A) Descriptive statistics for participants with dementia								
Number of people with dementia taking part at each year band (total = 1555)	860	943	851	394	327	190	112	70
Subtype (*N*, %)								
AD	431 (50.1%)	495 (52.5%)	475 (55.8%)	232 (58.9%)	207 (63.3%)	106 (55.8%)	74 (66.1%)	45 (64.3%)
VaD	84 (9.8%)	89 (9.4%)	77 (9.0%)	38 (9.6%)	31 (9.5%)	22 (11.6%)	13 (11.6%)	9 (12.9%)
Mixed AD/VaD	216 (25.1%)	240 (25.5%)	193 (22.7%)	75 (19.0%)	56 (17.1%)	37 (19.5%)	15 (13.4%)	8 (11.4%)
FTD	51 (5.9%)	45 (4.8%)	36 (4.2%)	15 (3.8%)	13 (4.0%)	12 (6.3%)	7 (6.3%)	5 (7.1%)
PDD	29 (3.4%)	32 (3.4%)	27 (3.2%)	17 (4.3%)	9 (2.8%)	9 (4.7%)	2 (1.8%)	2 (2.9%)
DLB	49 (5.7%)	42 (4.5%)	43 (5.1%)	17 (4.3%)	11 (3.4%)	4 (2.1%)	1 (0.9%)	1 (1.4%)
Sex (*N*, %)								
Male	459 (53.4%)	529 (56.1%)	481 (56.5%)	222 (56.3%)	181 (55.4%)	107 (56.3%)	62 (55.4%)	45 (64.3%)
Female	401 (46.6%)	414 (43.9%)	370 (43.5%)	172 (43.7%)	146 (44.6%)	83 (43.7%)	50 (44.6%)	25 (35.7%)
Age (*N*, %)								
< 65	88 (10.2%)	91 (9.7%)	84 (9.9%)	41 (10.4%)	27 (8.3%)	16 (8.4%)	9 (8.0%)	8 (11.4%)
65–69	91 (10.6%)	101 (10.7%)	72 (8.5%)	40 (10.2%)	36 (11.0%)	26 (13.7%)	13 (11.6%)	12 (17.1%)
70–74	140 (16.3%)	153 (16.2%)	150 (17.6%)	63 (16.0%)	59 (18.0%)	35 (18.4%)	21 (18.8%)	15 (21.4%)
75–79	192 (22.3%)	211 (22.4%)	186 (21.9%)	85 (21.6%)	72 (22.0%)	39 (20.5%)	17 (15.2%)	7 (10.0%)
80 +	349 (40.6%)	387 (41.0%)	359 (42.2%)	165 (41.9%)	133 (40.7%)	74 (38.9%)	52 (46.4%)	28 (40.0%)
Self-rated QoL-AD (mean (SD), *N*)	36.48 (5.89), 775	36.96 (5.98), 851	36.66 (5.82), 736	37.08 (5.76), 341	37.06 (6.13), 272	36.79 (5.35), 156	37.34 (5.33), 103	35.68 (5.73), 62
Diagnosis subtype	Self-rated QoL-AD (mean (SD), *N*)							
AD	37.47 (5.35), 390	37.98 (5.50), 452	37.61 (5.49), 417	37.72 (5.72), 197	37.05 (5.84), 174	37.44 (5.29), 85	38.51 (4.83), 67	36.46 (5.91), 39
VaD	34.69 (6.15), 74	35.99 (6.59), 74	36.70 (6.81), 60	38.82 (6.33), 33	36.50 (5.37), 24	37.11 (6.91), 19	34.17 (5.52), 12	37.11 (5.16), 9
Mixed AD/VaD	36.27 (5.83), 198	36.79 (5.63), 223	36.07 (5.58), 168	36.27 (4.88), 67	35.23 (6.44), 47	35.14 (4.71), 29	34.07 (5.24), 14	32.00 (3.56), 7
FTD	37.21 (6.95), 47	35.68 (6.73), 41	34.41 (5.75), 34	36.93 (5.54), 14	37.46 (5.92), 13	37.92 (4.50), 12	40.43 (4.43), 7	34.00 (6.82), 5
PDD	33.00 (6.04), 24	32.00 (7.25), 28	31.96 (6.48), 24	32.07 (6.19), 15	32.17 (5.53), 6	33.75 (4.65), 8	31.00 (5.66), 2	33.00, 1
DLB	32.64 (6.34), 42	32.09 (6.24), 33	33.18 (5.11), 33	33.73 (4.93), 15	31.00 (7.13), 8	36.00 (2.65), 3	34.00, 1	29.00, 1
(B) Descriptive statistics for informants								
Number of carers taking part at each year band (total = 1327)	692	772	741	364	291	193	134	79
Kin relationship (*N*, %)								
Spouse/partner	538 (77.7%)	626 (81.1%)	607 (81.9%)	306 (84.1%)	254 (87.3%)	173 (89.6%)	113 (84.3%)	70 (88.6%)
Family/friend	154 (22.3%)	146 (18.9%)	134 (18.1%)	58 (15.9%)	37 (12.7%)	20 (10.4%)	21 (15.7%)	9 (11.4%)
Informant-rated QoL-AD (mean (SD), *N*)	33.99 (5.96), 633	33.07 (5.87), 729	32.28 (5.88), 697	31.82 (5.97), 344	31.77 (6.17), 276	31.32 (5.72), 187	30.40 (6.34), 131	30.70 (6.49), 76
Diagnosis subtype	Informant-rated QoL-AD (mean (SD), *N*)							
AD	34.58 (5.66), 324	33.92 (5.79), 382	33.03 (5.68), 384	32.65 (5.82), 193	32.07 (5.95), 172	31.67 (5.58), 110	30.77 (6.57), 87	31.19 (6.74), 47
VaD	32.24 (6.92), 62	33.22 (5.51). 68	32.30 (5.88), 63	32.97 (6.15), 32	31.86 (6.58), 22	32.47 (5.49), 17	28.60 (4.38), 10	35.25 (4.68), 8
Mixed AD/VaD	34.01 (6.28), 148	32.43 (5.93), 178	31.67 (6.13), 154	30.95 (5.57), 66	30.88 (6.73), 50	29.85 (6.53), 34	29.38 (5.55), 21	28.22 (6.23), 9
FTD	35.00 (5.61), 38	32.22 (6.40), 37	32.34 (5.93), 32	28.84 (6.13), 19	32.42 (6.22), 12	31.82 (5.38), 11	32.50 (3.56), 6	29.14 (5.24), 7
PDD	31.56 (5.33), 27	29.14 (4.41), 29	30.19 (4.72), 27	30.53 (6.18), 17	30.89 (7.91), 9	29.50 (5.46), 10	33.25 (11.67), 4	28.00 (3.37), 4
DLB	32.26 (5.17), 34	30.91 (5.61), 35	28.59 (5.86), 37	28.24 (6.27), 17	31.00 (5.31), 11	32.20 (4.76), 5	25.00 (5.00), 3	23.00 (2.83), 2

*AD* Alzheimer’s disease, *VaD* Vascular dementia, *FTD* Frontotemporal dementia, *PDD* Parkinson’s disease dementia, *DLB* Dementia with Lewy bodies, *SD* Standard deviation, *N* Number of participants, *QoL-AD* Quality of Life in Alzheimer's Disease

### Self-rated quality of life

**Fig. 1:**
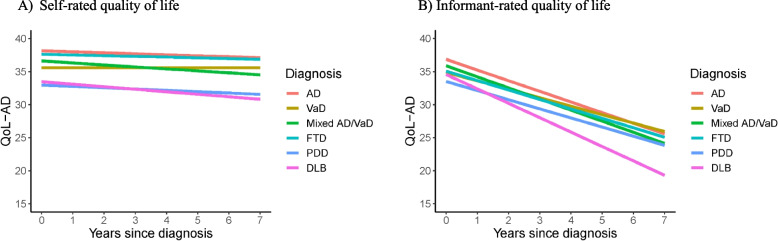
Trajectories of quality of life by diagnostic group. Notes: Figures are a visualisation of the results from model 2 presented in Table 2 (self-rated QoL) and Table 3 (informant-rated QoL). These models assume a linear trend. For self-rated quality of life model 2 is adjusted for age and sex, and for informant-rated quality of life model 2 is adjusted for age and sex of the person with dementia, and kin relationship; the figures are representative of the reference categories of each model; a person with dementia who is aged 80 + and male (**A**) and a person with dementia who is aged 80 + , male, and has a spouse informant (**B**). Graphs representing the unadjusted models are presented in Additional file 1: Fig. S1 for comparison

At the time of diagnosis (intercept), people with VaD, mixed dementia, PDD, or DLB had a lower estimated self-rated QoL-AD score than people with AD in the unadjusted model, the model adjusted for sex (model 1), and the model adjusted for age and sex (model 2); see Table 2 and Fig. 1A. There is some evidence that people with FTD had a lower estimated QoL-AD score compared to people with AD in the unadjusted model and in the model adjusted for sex, but since confidence intervals span zero there is less certainty in these findings. People with PDD or DLB had the lowest estimated QoL-AD scores of all diagnostic groups. Findings remained similar following further adjustment for cognition (model 3).

**Table 2:** Associations between diagnostic group and self-rated quality of life using a joint model

		AD	VaD	Mixed AD/VaD	FTD	PDD	DLB
Unadjusted	Intercept (estimate, 95% CI)	Ref	− 2.44 (− 3.64, − 1.23)	− 1.22 (− 2.09, − 0.35)	− 1.45 (− 2.99, 0.10)	− 5.03 (− 6.90, − 3.15)	− 4.57 (− 6.29, − 2.85)
	Slope (change per year) (estimate, 95% CI)	Ref	0.13 (− 0.16, 0.41)	− 0.20 (− 0.43, 0.04)	0.09 (− 0.19, 0.37)	− 0.07 (− 0.52, 0.39)	− 0.29 (− 0.86, 0.28)
Model 1	Intercept (estimate, 95% CI)	Ref	− 2.46 (− 3.65, − 1.27)	− 1.20 (− 2.07, − 0.34)	− 1.38 (− 2.87, 0.11)	− 5.13 (− 7.01, − 3.25)	− 4.64 (− 6.36, − 2.91)
	Slope (change per year) (estimate, 95% CI)	Ref	0.15 (− 0.14, 0.44)	− 0.21 (− 0.44, 0.03)	0.12 (− 0.17, 0.42)	− 0.05 (− 0.50, 0.40)	− 0.24 (− 0.81, 0.34)
Model 2	Intercept (estimate, 95% CI)	Ref	− 2.54 (− 3.77, − 1.31)	− 1.52 (− 2.40, − 0.65)	− 0.52 (− 1.88, 0.84)	− 5.17 (− 7.04, − 3.29)	− 4.66 (− 6.35, − 2.97)
	Slope (change per year) (estimate, 95% CI)	Ref	0.15 (− 0.18, 0.47)	− 0.15 (− 0.39, 0.09)	0.04 (− 0.28, 0.35)	− 0.05 (− 0.54, 0.43)	− 0.23 (− 0.78, 0.31)
Model 3	Intercept (estimate, 95% CI)	Ref	− 2.61 (− 3.85, − 1.38)	− 1.51 (− 2.37, − 0.65)	− 0.54 (− 1.84, 0.77)	− 5.10 (− 6.99, − 3.23)	− 4.73 (− 6.42, − 3.05)
	Slope (change per year) (estimate, 95% CI)	Ref	0.12 (− 0.18, 0.42)	− 0.14 (− 0.38, 0.09)	0.02 (− 0.28, 0.32)	− 0.11 (− 0.58, 0.37)	− 0.21 (− 0.78, 0.35)

Model 1 is adjusted for sex only. Model 2 is adjusted for age group and sex. Model 3 is adjusted for age group, sex, and cognition. Full details of the model are present in Additional file 1: Table S4a–d. *AD* Alzheimer’s disease, *VaD* Vascular dementia, *FTD* Frontotemporal dementia, *PDD* Parkinson’s disease dementia, *DLB* Dementia with Lewy bodies, *ref* Reference group, *CI* Confidence interval

Self-rated QoL-AD scores were generally stable over time (estimated decline of − 0.15 points per year, 95% CI − 0.38, 0.08, for someone with AD, male, and aged 80 + ; model 2); see Fig. 1A. There was little evidence of a meaningful difference in the trajectories of self-rated QoL-AD scores between any of the non-AD diagnostic groups and AD. All comparisons indicated small differences, with the largest estimated annual change being around a quarter of a point. Findings remained similar following further adjustment for cognition (model 3). The full details of the models are reported in Additional file 1: Table S4.

Sensitivity analysis incorporating data only up to 8 years post-diagnosis shows similar findings (Additional file 1: Table S1). Additionally, findings for self-rated SwLS and WHO-5 scores were similar to those for self-rated QoL-AD; see Additional file 1: Tables S5–6.

For the time-to-dropout model, there is evidence that people with mixed dementia, FTD, PDD, or DLB were more at risk of dropout compared to people with AD. However, there was limited evidence that dropout was associated with QoL-AD score in the joint model (hazard ratio: 0.99, 95% CI 0.97, 1.00; see Additional file 1: Table S4c). Mean QoL-AD scores are reported in Additional file 1: Table S2 for those who remain in the study at the next timepoint and those who dropped out. There was limited evidence that those who dropped out had lower QoL-AD scores, with numerical differences being small. However, there is some evidence that those who subsequently died had lower QoL-AD scores before they dropped out.

### Informant-rated quality of life

At the time of diagnosis (intercept), compared to people with AD, people with other dementia diagnoses had lower estimated informant-rated QoL-AD scores in all models; see Table 3 and Fig. 1B. Again, in models adjusted for age, sex, and kin relationship (model 2), and upon further adjustment for person with dementia cognition (model 3), people with PDD or DLB had the lowest estimated QoL-AD scores of all diagnostic groups. When comparing Fig. 1A and B, it is clear that differences between the groups at the time of diagnosis were less for informant-rated QoL-AD scores than for self-rated QoL-AD scores.

**Table 3:** Associations of diagnostic group and informant-rated quality of life using a joint model

		AD	VaD	Mixed AD/VaD	FTD	PDD	DLB
Unadjusted	Intercept (estimate, 95% CI)	Ref	− 2.30 (− 3.41, − 1.18)	− 1.13 (− 2.10, − 0.16)	− 2.02 (− 3.34, − 0.70)	− 2.86 (− 4.38, − 1.34)	− 1.88 (− 3.64, − 0.12)
	Slope (change per year) (estimate, 95% CI)	Ref	0.40 (0.13, 0.68)	− 0.17 (− 0.44, 0.11)	0.31 (0.05, 0.56)	0.10 (− 0.44, 0.61)	− 0.75 (− 1.32, − 0.17)
Model 1	Intercept (estimate, 95% CI)	Ref	− 2.28 (− 3.41, − 1.15)	− 0.84 (− 1.79, 0.10)	− 2.19 (− 3.52, − 0.87)	− 2.59 (− 4.35, − 0.83)	− 2.25 (− 4.00, − 0.49)
	Slope (change per year) (estimate, 95% CI)	Ref	0.39 (0.09, 0.68)	− 0.18 (− 0.45, 0.08)	0.33 (0.06, 0.59)	− 0.31 (− 0.68, 0.07)	− 0.72 (− 1.29, − 0.15)
Model 2	Intercept (estimate, 95% CI)	Ref	− 1.96 (− 3.07, − 0.85)	− 0.95 (− 1.91, 0.01)	− 1.77 (− 3.15, − 0.40)	− 3.34 (− 5.13, − 1.55)	− 2.28 (− 4.09, − 0.48)
	Slope (change per year) (estimate, 95% CI)	Ref	0.33 (0.06, 0.60)	− 0.07 (− 0.32, 0.18)	0.18 (− 0.10, 0.45)	0.23 (− 0.15, 0.60)	− 0.57 (− 1.16, 0.02)
Model 3	Intercept (estimate, 95% CI)	Ref	− 1.63 (− 3.01, − 0.26)	− 0.70 (− 1.65, 0.25)	− 1.22 (− 2.96, 0.53)	− 2.56 (− 4.36, − 0.76)	− 2.31 (− 4.10, − 0.51)
	Slope (change per year) (estimate, 95% CI)	Ref	0.13 (− 0.19, 0.46)	− 0.18 (− 0.44, 0.09)	0.03 (− 0.35, 0.41)	− 0.28 (− 0.73, 0.16)	− 0.18 (− 0.75, 0.38)

Model 1 is adjusted for sex and kin relationship. Model 2 is adjusted for age group, sex, and kin relationship. Model 3 is adjusted for age group, sex, kin relationship, and cognition. Full details of the model are present in Additional file 1: Table S7a–d

*CI* Confidence intervals, *Ref* Reference category, *AD* Alzheimer’s disease, *VaD* Vascular dementia, *FTD* Frontotemporal dementia, *PDD* Parkinson’s disease dementia, *DLB* Dementia with Lewy bodies

Informant-rated QoL-AD scores declined over time (estimated decline of − 1.61 points per year, 95% CI − 1.93, − 1.30 for someone with AD, male, aged 80 + , and a spouse informant; model 2); see Fig. 1B. Compared with AD, there was little evidence of a meaningful difference between the trajectories of informant-rated QoL-AD scores for people with mixed dementia, FTD, or PDD. Scores for people with VaD, however, declined to a lesser extent (− 1.28 points per year for model 2). Scores for people with DLB declined to the greatest extent (− 2.18 points per year in model 2); although estimates approached statistical significance when compared with AD, QoL-AD scores for DLB declined significantly more compared with VaD (− 0.90 points per year, 95% CI − 1.53, − 0.27). Following adjustment for person with dementia cognition, differences between dementia types were attenuated. The full details of the models are reported in Additional file 1: Table S7.

Findings were similar for informant-rated quality of life in the sensitivity analyses incorporating data only up to 8 years post-diagnosis (Additional file 1: Table S1). Findings for informant-rated SwLS and WHO-5 scores were also similar to the findings presented for informant-rated QoL-AD; see Additional file 1: Tables S8–9.

For the time-to-dropout model, people with mixed dementia, PDD, or DLB were more at risk of dropout than people with AD and there was evidence of an association between dropout and lower scores on informant-rated QoL-AD in the joint model (hazard ratio: 0.92, 95% CI 0.91, 0.93; see Additional file 1: Table S7c).

## Discussion

This study investigated trajectories of scores for quality of life in people with the six most common types of dementia from the time of diagnosis and was made possible by the large size of the IDEAL cohort. Overall, self-ratings showed a small decline but were relatively stable over time for all diagnostic groups. The findings from our earlier studies were upheld as people with PDD or DLB reported the lowest self-rated quality of life scores of all diagnostic groups [7, 27]; the present study has extended this by showing that the trajectories for these two diagnostic groups remain consistently lower than those of other diagnostic groups over 6 years. The present study additionally investigated, for the first time, trajectories of informant-rated quality of life scores in the same six diagnostic groups. At the time of diagnosis, informant-rated quality of life was higher for AD than for all other diagnostic groups, and as with the self-ratings, people with PDD or DLB were rated as having the lowest quality of life. However, at the time of diagnosis, mean self-rated scores had a greater range across the subtypes than informant-rated scores. Informant-rated quality of life showed a steeper decline over the years following diagnosis than the relatively stable self-ratings for all six diagnostic groups, with the mean decrease ranging from 1.3 points per year for VaD to 2.2 points per year for DLB. As more time elapsed following diagnosis, informant ratings showed a degree of decline generally not seen in self-ratings, particularly for people with DLB.

The finding that self-rated quality of life was largely stable over time is consistent with our previous study [7] and extends this observation over a longer time period. The relatively stable trajectories of self-rated scores across types of dementia suggests that people with any form of dementia who already have low scores around the time of diagnosis are likely to continue to have low scores over time. This is broadly consistent with studies in people with prodromal dementia symptoms, such as subjective cognitive difficulties or mild cognitive impairment, who self-report lower quality of life scores than healthy older people [40–44]. Identifying people whose quality of life is poor as part of the diagnostic process could help target those most in need of additional help and support. The findings also highlight that compared to AD, quality of life scores were lower in all other diagnostic groups. Scores were particularly low for people with PDD or DLB; this may be due to concomitant difficulties primarily associated with these dementia types such as movement disorders or hallucinations, or autonomic symptoms including incontinence, falls, and sleep disorders [24]. Providing support that addresses the unique challenges of people with less common dementia types could help to improve their quality of life. Indeed, people in each diagnostic group want more tailored and specific support services that address the multiplicity of their symptoms [45].

The finding that informant-rated quality of life scores were lower than self-ratings at all timepoints is consistent with most previous studies [9], although in the present study mean self- and informant-rated QoL-AD scores for people with DLB were estimated as being very similar at the time of diagnosis, which contrasts with previous research [23]. Trajectories of informant-rated quality of life scores show a steeper decline than the corresponding self-ratings. Informant ratings are likely to be influenced by the cognitive ability of people with dementia as after controlling for cognitive ability, differences between diagnostic groups were attenuated. This is broadly consistent with cross sectional findings where greater cognitive difficulties have a larger effect on carer informant ratings than person with dementia self-ratings [9]. Increasing cognitive difficulties are associated with further difficulties with everyday functioning, challenges to communication and behaviour, and increased carer stress [28, 46]. This suggests that, for carers, cognitive decline and concomitant changes may have a stronger effect than the constellation of symptoms pertaining to the specific diagnosis of the cared-for person on their informant ratings of quality of life. Future research could investigate which factors, in addition to cognition, are associated with declining informant-rated quality of life.

This study has several limitations. Whilst attrition is to be expected within longitudinal research, especially where participants have progressive neurodegenerative conditions, the level of attrition over the 6 years of data collection was a major limitation. A joint model was used to attempt to compensate for this attrition, which enabled investigation of longitudinal outcomes whilst controlling for dropout. However, for self-rated QoL-AD, there was little evidence of an association between dropout and quality of life, which may be because quality of life mostly remains stable or because dropout was linked to other factors such as cognition or functional changes [28]. It may be that those who died had poorer QoL-AD scores, but since death is not well-recorded in the study it is difficult to draw conclusions about this. For those where death was recorded, there was some evidence that they had lower QoL-AD scores at the timepoint before they dropped out and therefore the stable self-rated QoL-AD scores over time could be attributable to healthy survivor bias. According to the joint model, there was evidence of a link between dropout and informant-rated quality of life. There were other factors contributing to dropout; for example, there was a 2-year gap in data collection between timepoints 3 and 4 and a high proportion of dropout occurred at this stage, and the COVID-19 pandemic disrupted data collection in the latter stages of timepoint 4 and throughout timepoints 5 and 6. Another limitation was the relatively small sample sizes for the less common types of dementia, particularly at later timepoints, even though the numbers in these groups were larger at baseline than in most other studies that have investigated quality of life [9]. In future research, it would be helpful to find ways of including larger numbers of people in these diagnostic groups to strengthen the generalisability of the findings. A strength of the study was the relatively long follow-up period, which meant that the present study could investigate changes that other studies using shorter timeframes might miss.

## Conclusions

This study provides valuable insights into the longitudinal trajectories of quality of life across dementia diagnoses. Most people with dementia, regardless of their specific diagnosis, perceived little change in quality of life over time. Instead, perceptions of quality of life may already be established at the time of diagnosis. Conversely, informant ratings showed a clear decline, which could be due to observable changes in cognition. People with PDD or DLB are particularly likely to score poorly on quality of life, suggesting that more consideration should be given to the reasons for this and possible mitigations. Exploring perceptions of quality of life as part of routine assessments could help identify changes and support needs. Further research is needed to identify ways of improving the long-term quality of life of people with dementia. By addressing these challenges, it may be possible to enhance post-diagnostic support and ultimately improve the overall well-being of people living with dementia.

### Supplementary Information

Additional file 1. Reasons for dropout and associations of dementia type with quality of life, satisfaction with life and well-being (.pdf): Table S1: Sensitivity analysis using only data from 0 to 8 years post-diagnosis showing associations of diagnostic group and self-rated or informant-rated quality of life using a joint model; Table S2: Reasons for dropout and non-participation during the study, and comparison of QoL-AD scores for those that dropped out and those that did not at the following year band; Table S3: Descriptive statistics for quality of life, satisfaction with life and well-being for participants with dementia; Table S4: Joint model for self-rated quality of life; Table S5: Joint model for self-rated satisfaction with life; Table S6: Joint model for self-rated well-being; Table S7: Joint model for informant-rated quality of life; Table S8: Joint model for informant-rated satisfaction with life; Table S9: Joint model for informant-rated well-being; Fig. S1: Trajectories of quality of life by diagnostic group

## Data Availability

IDEAL data were deposited with the UK data archive in April 2020. Details of how to access the data can be found here: https://reshare.ukdataservice.ac.uk/854293/.

## Abbreviations

AD: Alzheimer’s disease
CI: Confidence intervals
DLB: Dementia with Lewy bodies
FTD: Frontotemporal dementia
IDEAL: Improving the experience of Dementia and Enhancing Active Life
IDEAL-2: Improving the experience of Dementia and Enhancing Active Life-2
mixed dementia: Mixed Alzheimer’s and vascular dementia
MMSE: Mini-Mental State Examination
*N*: Number of participants
PDD: Parkinson’s disease dementia
QoL: Quality of life
QoL-AD: Quality of Life in AD
Ref: Reference category
SD: Standard deviation
SwLS: Satisfaction with Life Scale
UKCRN: UK Clinical Research Network
VaD: Vascular dementia
WHO-5: World Health Organization-Five Well-Being Index
