# Supplementary material for: Differences in trajectories of quality of life according to type of dementia: 6-year longitudinal findings from the IDEAL programme
Source: BMC Med. 2024 Jun 24;22:265. doi: 10.1186/s12916-024-03492-y (PMC11197262; doi:10.1186/s12916-024-03492-y)
Supplement: Supplementary file 1 — Additional file 1. Reasons for dropout and associations of dementia type with quality of life, satisfaction with life and well-being (.pdf): Table S1: Sensitivity analysis using only data from 0 to 8 years post-diagnosis showing associations of diagnostic group and self-rated or informant-rated quality of life using a joint model; Table S2: Reasons for dropout and non-participation during the study, and comparison of QoL-AD scores for those that dropped out and those that did not at the following year band; Table S3: Descriptive statistics for quality of life, satisfaction with life and well-being for participants with dementia; Table S4: Joint model for self-rated quality of life; Table S5: Joint model for self-rated satisfaction with life; Table S6: Joint model for self-rated well-being; Table S7: Joint model for informant-rated quality of life; Table S8: Joint model for informant-rated satisfaction with life; Table S9: Joint model for informant-rated well-being; Fig. S1: Trajectories of quality of life by diagnostic group [file 12916_2024_3492_MOESM1_ESM.docx]

Differences in trajectories of quality of life according to type of dementia: 6-year longitudinal findings from the IDEAL programme

Additional file 1: Reasons for dropout and associations of dementia type with quality of life, satisfaction with life and well-being.

Table S1. Sensitivity analysis using only data from 0-8 years post-diagnosis showing associations of diagnostic group and self-rated or informant-rated quality of life using a joint model.

1. Self-rated quality of life

|  | Unadjusted | | Model 1 | | Model 2 | | Model 3 | |
| --- | --- | --- | --- | --- | --- | --- | --- | --- |
|  | Intercept  (estimate, 95% CI) | Slope (change per year)  (estimate, 95% CI) | Intercept  (estimate, 95% CI) | Slope (change per year)  (estimate, 95% CI) | Intercept  (estimate, 95% CI) | Slope (change per year)  (estimate, 95% CI) | Intercept  (estimate, 95% CI) | Slope (change per year)  (estimate, 95% CI) |
| AD | Ref | Ref | Ref | Ref | Ref | Ref | Ref | Ref |
| VaD | -2.59 (-3.83, -1.36) | 0.21 (-0.12, 0.54) | -2.60 (-3.83, -1.37) | 0.21 (-0.12, 0.55) | -2.70 (-3.92, -1.49) | 0.23 (-0.11, 0.56) | -2.79 (-4.00, -1.59) | 0.17 (-0.16, 0.51) |
| Mixed AD/VaD | -1.26 (-2.15, -0.36) | -0.17 (-0.41, 0.08) | -1.28 (-2.17, -0.38) | -0.17 (-0.41, 0.08) | -1.59 (-2.48, -0.69) | -0.13 (-0.38, 0.13) | -1.56 (-2.43, -0.69) | -0.14 (-0.39, 0.11) |
| FTD | -1.36 (-2.87, 0.14) | -0.04 (-0.47, 0.40) | -1.35 (-2.87, 0.18) | 0.00 (-0.44, 0.45) | -0.38 (-1.84, 1.08) | -0.01 (-0.44, 0.41) | -0.42 (-1.85, 1.01) | -0.02 (-0.45, 0.41) |
| PDD | -4.94 (-6.93, -2.95) | -0.07 (-0.66, 0.53) | -4.96 (-6.96, -2.97) | -0.08 (-0.67, 0.51) | -5.04 (-7.04, -3.05) | -0.06 (-0.66, 0.53) | -5.02 (-7.01, -3.02) | -0.12 (-0.72, 0.49) |
| DLB | -4.62 (-6.36, -2.88) | -0.27 (-0.85, 0.32) | -4.64 (-6.38, -2.90) | -0.24 (-0.81, 0.33) | -4.65 (-6.34, -2.96) | -0.23 (-0.78, 0.33) | -4.72 (-6.39, -3.04) | -0.21 (-0.79, 0.37) |

1. Informant-rated quality of life

|  | Unadjusted | | Model 1 | | Model 2 | | Model 3 | |
| --- | --- | --- | --- | --- | --- | --- | --- | --- |
|  | Intercept  (estimate, 95% CI) | Slope (change per year)  (estimate, 95% CI) | Intercept  (estimate, 95% CI) | Slope (change per year)  (estimate, 95% CI) | Intercept  (estimate, 95% CI) | Slope (change per year)  (estimate, 95% CI) | Intercept  (estimate, 95% CI) | Slope (change per year)  (estimate, 95% CI) |
| AD | Ref | Ref | Ref | Ref | Ref | Ref | Ref | Ref |
| VaD | -2.20 (-3.38, -1.01) | 0.41 (0.04, 0.79) | -2.20 (-3.37, -1.02) | 0.46 (0.07, 0.84) | -2.01 (-3.17, -0.85) | 0.40 (0.01, 0.79) | -1.92 (-3.22, -0.62) | 0.26 (-0.12, 0.65) |
| Mixed AD/VaD | -1.03 (-1.99, -0.06) | -0.13 (-0.43, 0.17) | -0.71 (-1.66, 0.25) | -0.15 (-0.45, 0.15) | -0.66 (-1.67, 0.35) | -0.16 (-0.44, 0.12) | -0.72 (-1.68, 0.23) | -0.16 (-0.44, 0.12) |
| FTD | -1.24 (-2.70, 0.22) | -0.01 (-0.45, 0.44) | -1.37 (-2.82, 0.08) | 0.03 (-0.40, 0.47) | -1.05 (-2.55, 0.45) | -0.16 (-0.62, 0.30) | -1.04 (-2.72, 0.64) | -0.08 (-0.60, 0.43) |
| PDD | -2.53 (-4.35, -0.70) | 0.18 (-0.68, 0.32) | -2.86 (-4.67, -1.04) | -0.11 (-0.60, 0.38) | -3.27 (-5.07, -1.47) | 0.05 (-0.47, 0.58) | -2.82 (-4.77, -0.88) | -0.21 (-0.78, 0.37) |
| DLB | -1.92 (-3.64, -0.20) | -0.68 (-1.28, -0.09) | -2.25 (-3.97, -0.52) | -0.60 (-1.20, -0.00) | -2.28 (-3.97, -0.58) | -0.60 (-1.21, 0.02) | -2.33 (-4.17, -0.49) | -0.23 (-0.82, 0.35) |

Notes: CI, confidence intervals. Ref, reference category; AD, Alzheimer’s disease; VaD, vascular dementia; FTD, frontotemporal dementia; PDD, Parkinson’s disease dementia; DLB, dementia with Lewy bodies. A) Model 1 is adjusted for sex. Model 2 is adjusted for age group and sex. Model 3 is adjusted for age group, sex, and MMSE. B) Model 1 is adjusted for sex and kin relationship. Model 2 is adjusted for age group, sex, and kin relationship. Model 3 is adjusted for age group, sex, kin relationship, and MMSE.

Table S2. Reasons for dropout and non-participation during the study, and comparison of QoL-AD scores for those that dropped out and those that did not at the following year band.

|  | Years since diagnosis | | | | | | | |
| --- | --- | --- | --- | --- | --- | --- | --- | --- |
|  | <1 | 1 to <2 | 2 to <3 | 3 to <4 | 4 to <5 | 5 to <6 | 6 to <7 | 7 to <8 |
| Number taking part during this year band | 860 | 943 | 851 | 394 | 327 | 190 | 112 | 70 |
| Died | - | 35 | 49 | 95 | 40 | 38 | 16 | 9 |
| Withdrew: health reasons | - | 43 | 73 | 54 | 37 | 27 | 17 | 8 |
| Withdrew: not interested | - | 77 | 75 | 135 | 60 | 64 | 30 | 16 |
| Withdrew: moved into residential care | - | 4 | 6 | 8 | 2 | 12 | 10 | 1 |
| Withdrew: lost to follow-up | - | 57 | 59 | 112 | 41 | 47 | 20 | 10 |
| Did not take part at this timepoint (intermittent) | - | 41 | 37 | 158 | 94 | 66 | 46 | 25 |
|  |  |  |  |  |  |  |  |  |
| QoL-AD scores for participants who: |  |  |  |  |  |  |  |  |
| Remained in the study at the next year band | 36.7 (5.8) | 37.3 (5.9) | 37.3 (5.5) | 37.2 (5.5) | 38.0 (5.8) | 37.3 (6.0) | 37.5 (5.5) | 36.7 (3.7) |
| Dropped out at the next year band (exc. death) | 36.0 (6.1) | 36.4 (6.0)* | 36.4 (6.0)* | 37.8 (5.8) | 36.9 (5.9) | 36.0 (4.7) | 37.0 (3.9) | 34.9 (6.6) |
| Dropped out at the next year band (inc. death) | 35.9 (6.1) | 35.9 (6.0)* | 36.0 (6.1)* | 37.0 (6.1) | 36.2 (6.3)* | 36.2 (4.9) | 37.3 (4.1) | 35.3 (6.2) |
| Died before the next year band | 35.2 (6.0) | 33.8 (5.8)* | 34.3 (6.2)* | 34.0 (6.1)* | 33.6 (7.2)* | 38.0 (5.6) | 37.0 (5.5) | 36.2 (5.6) |

* P < 0.05 in an unpaired two-samples t-test, with comparison group ‘remained in study at next year band’. Dropout includes health reasons, not interested, moved into residential care, lost to follow up, and died where specified.

Table S3. Descriptive statistics for quality of life, satisfaction with life and well-being for participants with dementia

1. Self-rated measures

|  | Years since diagnosis | | | | | | | |
| --- | --- | --- | --- | --- | --- | --- | --- | --- |
|  | <1 | 1 to <2 | 2 to <3 | 3 to <4 | 4 to <5 | 5 to <6 | 6 to <7 | 7 to <8 |
| Number of people with dementia taking part at each year band (total = 1555) | 860 | 943 | 851 | 394 | 327 | 190 | 112 | 70 |
| QoL-AD  mean (SD),  N (%) | 36.48 (5.89), 775 (90.1%) | 36.96 (5.98), 851 (90.2%) | 36.66 (5.82), 736 (86.5%) | 37.08 (5.76), 341 (86.5%) | 37.06 (6.13), 272 (83.2%) | 36.79 (5.35), 156 (82.1%) | 37.34 (5.33), 103 (92.0%) | 35.68 (5.73), 62 (88.6%) |
| SwLS  mean (SD),  N (%) | 25.65 (6.35),  838 (97.4%) | 26.07 (6.06),  897 (95.1%) | 25.98 (6.25),  781 (91.8%) | 26.10 (6.36),  364 (92.4%) | 26.71 (6.03),  290 (88.7%) | 26.68 (5.78),  168 (88.9%) | 26.01 (6.61),  106 (94.6%) | 25.50 (6.21),  62 (88.6%) |
| WHO-5  mean (SD),  N (%) | 59.53 (20.82), 850 (98.8%) | 60.63 (21.08), 912 (96.7%) | 60.12 (21.09), 800 (94.0%) | 60.35 (21.08), 369 (93.7%) | 62.39 (20.53), 315 (96.3%) | 60.54 (21.63), 177 (93.2%) | 63.66 (20.85), 107 (95.5%) | 57.94 (21.05), 68 (97.1%) |

1. Informant-rated measure

|  | Years since diagnosis | | | | | | | |
| --- | --- | --- | --- | --- | --- | --- | --- | --- |
|  | <1 | 1 to <2 | 2 to <3 | 3 to <4 | 4 to <5 | 5 to <6 | 6 to <7 | 7 to <8 |
| Number of carers taking part at each year band  (total = 1327) | 692 | 772 | 741 | 364 | 291 | 193 | 134 | 79 |
| QoL-AD  mean (SD),  N (%) | 33.99 (5.96), 633 (91.5%) | 33.07 (5.87), 729 (94.4%) | 32.28 (5.88), 697 (94.1%) | 31.82 (5.97), 344 (94.5%) | 31.77 (6.17), 276 (94.8%) | 31.32 (5.72), 187 (96.9%) | 30.40 (6.34), 131 (97.8%) | 30.70 (6.49), 76 (96.2%) |
| SwLS  mean (SD),  N (%) | 21.25 (6.96),  665 (96.1%) | 20.53 (7.01),  761 (98.6%) | 19.98 (7.19),  728 (98.2%) | 19.37 (7.26),  351 (96.4%) | 20.32 (7.08),  284 (97.6%) | 19.44 (7.05),  191 (99.0%) | 19.27 (7.76),  132 (98.5%) | 18.19 (7.42),  77 (97.5%) |
| WHO-5  mean (SD),  N (%) | 50.02 (20.73),  665 (96.1%) | 48.90 (20.41),  750 (97.2%) | 48.24 (21.09),  724 (97.7%) | 45.80 (20.39),  356 (97.8%) | 48.04 (20.79),  284 (97.6%) | 47.20 (18.99),  190 (98.4%) | 44.30 (22.47),  132 (98.5%) | 40.47 (21.20),  77 (97.5%) |

Table S4. Joint model for self-rated quality of life

1. Unadjusted model

|  | Linear Mixed Effects Model | | Survival Model | Joint Model | | |
| --- | --- | --- | --- | --- | --- | --- |
|  | Intercept  (estimate, 95% CI) | Slope (change per year)  (estimate, 95% CI) | Survival  (HR, 95% CI) | Intercept  (estimate, 95% CI) | Slope (change per year)  (estimate, 95% CI) | Survival  (HR, 95% CI) |
| AD | Ref | Ref | Ref | Ref | Ref | Ref |
| VaD | -2.35 (-3.54, -1.17) | 0.16 (-0.13, 0.45) | 1.02 (0.85, 1.23) | -2.44 (-3.64, -1.23) | 0.13 (-0.16, 0.41) | 0.99 (0.82, 1.19) |
| Mixed AD/VaD | -1.18 (-2.03, -0.33) | -0.19 (-0.42, 0.04) | 1.30 (1.14, 1.48) | -1.22 (-2.09, -0.35) | -0.20 (-0.43, 0.04) | 1.27 (1.10, 1.45) |
| FTD | -1.34 (-2.78, 0.11) | 0.16 (-0.18, 0.50) | 1.01 (0.79, 1.29) | -1.45 (-2.99, 0.10) | 0.09 (-0.19, 0.37) | 0.97 (0.76, 1.24) |
| PDD | -5.07 (-6.93, -3.22) | -0.02 (-0.51, 0.47) | 1.19 (0.91, 1.55) | -5.03 (-6.90, -3.15) | -0.07 (-0.52, 0.39) | 1.10 (0.83, 1.45) |
| DLB | -4.71 (-6.40, -3.01) | -0.13 (-0.73, 0.46) | 1.65 (1.31, 2.09) | -4.57 (-6.29, -2.85) | -0.29 (-0.86, 0.28) | 1.55 (1.21, 1.98) |
| Association | | | | | | 0.99 (0.97, 1.00) |

1. Model 1: Adjusted for sex

|  | Linear Mixed Effects Model | | Survival Model | Joint Model | | |
| --- | --- | --- | --- | --- | --- | --- |
|  | Intercept  (estimate, 95% CI) | Slope (change per year)  (estimate, 95% CI) | Survival  (HR, 95% CI) | Intercept  (estimate, 95% CI) | Slope (change per year)  (estimate, 95% CI) | Survival  (HR, 95% CI) |
| AD | Ref | Ref | Ref | Ref | Ref | Ref |
| VaD | -2.35 (-3.54, -1.17) | 0.16 (-0.13, 0.45) | 1.02 (0.85, 1.23) | -2.46 (-3.65, -1.27) | 0.15 (-0.14, 0.44) | 1.00 (0.83, 1.21) |
| Mixed AD/VaD | -1.18 (-2.03, -0.33) | -0.19 (-0.42, 0.04) | 1.30 (1.14, 1.49) | -1.20 (-2.07, -0.34) | -0.21 (-0.44, 0.03) | 1.28 (1.12, 1.47) |
| FTD | -1.34 (-2.78, 0.11) | 0.16 (-0.18, 0.50) | 1.02 (0.79, 1.30) | -1.38 (-2.87, 0.11) | 0.12 (-0.17, 0.42) | 0.99 (0.77, 1.27) |
| PDD | -5.07 (-6.93, -3.22) | -0.02 (-0.51, 0.47) | 1.19 (0.92, 1.56) | -5.13 (-7.01, -3.25) | -0.05 (-0.50, 0.40) | 1.15 (0.87, 1.51) |
| DLB | -4.71 (-6.40, -3.01) | -0.13 (-0.73, 0.46) | 1.67 (1.32, 2.11) | -4.64 (-6.36, -2.91) | -0.24 (-0.81, 0.34) | 1.60 (1.25, 2.05) |
| Sex | 0.26 (-0.43, 0.95) | -0.17 (-0.34, 0.01) | 0.96 (0.86, 1.07) | 0.19 (-0.50, 0.88) | -0.17 (-0.34, 0.01) | 0.97 (0.87, 1.08) |
| Association | | | | | | 0.99 (0.97, 1.00) |

1. Model 2: Adjusted for sex and age group

|  | Linear Mixed Effects Model | | Survival Model | Joint Model | | |
| --- | --- | --- | --- | --- | --- | --- |
|  | Intercept  (estimate, 95% CI) | Slope (change per year)  (estimate, 95% CI) | Survival  (HR, 95% CI) | Intercept  (estimate, 95% CI) | Slope (change per year)  (estimate, 95% CI) | Survival  (HR, 95% CI) |
| AD | Ref | Ref | Ref | Ref | Ref | Ref |
| VaD | -2.42 (-3.59, -1.25) | 0.15 (-0.14, 0.44) | 1.06 (0.88, 1.27) | -2.54 (-3.77, -1.31) | 0.15 (-0.18, 0.47) | 1.03 (0.86, 1.25) |
| Mixed AD/VaD | -1.49 (-2.34, -0.64) | -0.15 (-0.39, 0.08) | 1.21 (1.06, 1.39) | -1.52 (-2.40, -0.65) | -0.15 (-0.39, 0.09) | 1.18 (1.03, 1.36) |
| FTD | -0.53 (-2.01, 0.94) | 0.06 (-0.29, 0.41) | 1.35 (1.04, 1.75) | -0.52 (-1.88, 0.84) | 0.04 (-0.28, 0.35) | 1.31 (1.01, 1.69) |
| PDD | -5.14 (-6.99, -3.28) | -0.01 (-0.50, 0.48) | 1.30 (0.99, 1.70) | -5.17 (-7.04, -3.29) | -0.05 (-0.54, 0.43) | 1.20 (0.90, 1.58) |
| DLB | -4.75 (-6.43, -3.06) | -0.12 (-0.71, 0.47) | 1.76 (1.39, 2.23) | -4.66 (-6.35, -2.97) | -0.23 (-0.78, 0.31) | 1.64 (1.28, 2.10) |
| Sex | 0.26 (-0.42, 0.94) | -0.16 (-0.34, 0.02) | 0.97 (0.87, 1.08) | 0.15 (-0.53, 0.83) | -0.17 (-0.34, 0.01) | 0.98 (0.88, 1.09) |
| Age <65 | -2.14 (-3.23, -1.05) | 0.25 (-0.02, 0.53) | 0.45 (0.38, 0.54) | -2.17 (-3.26, -1.07) | 0.30 (0.02, 0.59) | 0.45 (0.38, 0.54) |
| Age 65-69 | -0.45 (-1.59, 0.69) | 0.08 (-0.22, 0.37) | 0.50 (0.42, 0.60) | -0.44 (-1.56, 0.67) | 0.17 (-0.12, 0.46) | 0.52 (0.43, 0.62) |
| Age 70-74 | 0.04 (-0.96, 1.04) | 0.02 (-0.28, 0.31) | 0.71 (0.61, 0.82) | 0.16 (-0.87, 1.18) | 0.08 (-0.21, 0.36) | 0.72 (0.62, 0.84) |
| Age 75-79 | 0.58 (-0.36, 1.52) | -0.03 (-0.30, 0.24) | 0.70 (0.60, 0.81) | 0.68 (-0.27, 1.62) | 0.02 (-0.25, 0.28) | 0.71 (0.62, 0.83) |
| Age 80+ | Ref | Ref | Ref | Ref | Ref | Ref |
| Association | | | | | | 0.99 (0.97, 1.00) |

1. Model 3: Adjusted for sex, age group, and MMSE

|  | Linear Mixed Effects Model | | Survival Model | Joint Model | | |
| --- | --- | --- | --- | --- | --- | --- |
|  | Intercept  (estimate, 95% CI) | Slope (change per year)  (estimate, 95% CI) | Survival  (HR, 95% CI) | Intercept  (estimate, 95% CI) | Slope (change per year)  (estimate, 95% CI) | Survival  (HR, 95% CI) |
| AD | Ref | Ref | Ref | Ref | Ref | Ref |
| VaD | -2.42 (-3.59, -1.25) | 0.11 (-0.18, 0.41) | 1.07 (0.88, 1.28) | -2.61 (-3.85, -1.38) | 0.12 (-0.18, 0.42) | 1.05 (0.87, 1.27) |
| Mixed AD/VaD | -1.47 (-2.31, -0.62) | -0.16 (-0.40, 0.07) | 1.22 (1.07, 1.40) | -1.51 (-2.37, -0.65) | -0.14 (-0.39, 0.09) | 1.21 (1.05, 1.38) |
| FTD | -0.59 (-2.07, 0.88) | 0.05 (-0.31, 0.40) | 1.40 (1.08, 1.82) | -0.54 (-1.84, 0.77) | 0.02 (-0.28, 0.32) | 1.35 (1.04, 1.76) |
| PDD | -5.06 (-6.91, -3.20) | -0.05 (-0.55, 0.44) | 1.28 (0.98, 1.67) | -5.11 (-6.99, -3.23) | -0.11 (-0.54, 0.37) | 1.22 (0.92, 1.61) |
| DLB | -4.76 (-6.44, -3.08) | -0.11 (-0.70, 0.49) | 1.77 (1.40, 2.24) | -4.73 (-6.42, -3.05) | -0.21 (-0.78, 0.35) | 1.70 (1.32, 2.18) |
| Sex | 0.20 (-0.48, 0.88) | -0.17 (-0.35, 0.01) | 0.99 (0.89, 1.10) | 0.05 (-0.62, 0.71) | -0.17 (-0.34, -0.00) | 1.00 (0.89, 1.11) |
| Age <65 | -2.12 (-3.21, -1.03) | 0.20 (-0.08, 0.49) | 0.47 (0.39, 0.56) | -2.12 (-3.22, -1.02) | 0.25 (0.02, 0.53) | 0.48 (0.39, 0.56) |
| Age 65-69 | -0.47 (-1.62, 0.67) | 0.04 (-0.26, 0.34) | 0.51 (0.43, 0.61) | -0.49 (-1.58, 0.60) | 0.14 (-0.12, 0.43) | 0.52 (0.44, 0.63) |
| Age 70-74 | -0.01 (-1.01, 1.00) | 0.02 (-0.27, 0.32) | 0.71 (0.61, 0.83) | 0.16 (-0.84, 1.15) | 0.07 (-0.21, 0.34) | 0.72 (0.62, 0.84) |
| Age 75-79 | 0.54 (-0.40, 1.47) | -0.02 (-0.29, 0.25) | 0.70 (0.61, 0.81) | 0.69 (-0.25, 1.62) | 0.03 (-0.25, 0.30) | 0.72 (0.62, 0.83) |
| Age 80+ | Ref | Ref | Ref | Ref | Ref | Ref |
| MMSE | 0.07 (0.00, 0.13) | 0.00 (-0.01, 0.02) | 0.97 (0.96, 0.99) | 0.06 (-0.01, 0.12) | 0.00 (-0.01, 0.02) | 0.98 (0.97, 0.99) |
| Association | | | | | | 0.98 (0.97, 1.00) |

Notes: HR, hazard ratio; CI, confidence intervals; AD, Alzheimer’s disease; VaD, vascular dementia; FTD, frontotemporal dementia; PDD, Parkinson’s disease dementia; DLB, dementia with Lewy bodies; MMSE, Mini-Mental State Examination; Ref, reference category.

Table S5. Joint model for self-rated satisfaction with life

1. Unadjusted model

|  | Linear Mixed Effects Model | | Survival Model | Joint Model | | |
| --- | --- | --- | --- | --- | --- | --- |
|  | Intercept  (estimate, 95% CI) | Slope (change per year)  (estimate, 95% CI) | Survival  (HR, 95% CI) | Intercept  (estimate, 95% CI) | Slope (change per year)  (estimate, 95% CI) | Survival  (HR, 95% CI) |
| AD | Ref | Ref | Ref | Ref | Ref | Ref |
| VaD | -1.68 (-2.91, -0.45) | -0.04 (-0.36, 0.29) | 1.03 (0.86, 1.23) | -1.69 (-2.93, -0.46) | -0.02 (-0.37, 0.33) | 1.01 (0.84, 1.21) |
| Mixed AD/VaD | -0.70 (-1.59, 0.19) | -0.01 (-0.27, 0.25) | 1.29 (1.13, 1.47) | -0.70 (-1.60, 0.19) | 0.02 (-0.23, 0.28) | 1.28 (1.12, 1.46) |
| FTD | -2.58 (-4.10, -1.07) | 0.16 (-0.22, 0.55) | 1.02 (0.80, 1.30) | -2.72 (-4.14, -1.29) | 0.23 (-0.09, 0.55) | 0.98 (0.77, 1.25) |
| PDD | -4.52 (-6.42, -2.61) | -0.16 (-0.69, 0.38) | 1.21 (0.93, 1.57) | -4.48 (-6.42, -2.54) | -0.17 (-0.70, 0.37) | 1.17 (0.89, 1.53) |
| DLB | -4.45 (-6.22, -2.68) | 0.09 (-0.58, 0.74) | 1.66 (1.32, 2.09) | -4.42 (-6.23, -2.60) | 0.11 (-0.54, 0.76) | 1.62 (1.27, 2.05) |
| Association | | | | | | 0.99 (0.98, 1.01) |

1. Model 1: Adjusted for sex

|  | Linear Mixed Effects Model | | Survival Model | Joint Model | | |
| --- | --- | --- | --- | --- | --- | --- |
|  | Intercept  (estimate, 95% CI) | Slope (change per year)  (estimate, 95% CI) | Survival  (HR, 95% CI) | Intercept  (estimate, 95% CI) | Slope (change per year)  (estimate, 95% CI) | Survival  (HR, 95% CI) |
| AD | Ref | Ref | Ref | Ref | Ref | Ref |
| VaD | -1.72 (-2.95, -0.48) | -0.03 (-0.35, 0.30) | 1.03 (0.86, 1.24) | -1.72 (-2.97, -0.46) | -0.01 (-0.39, 0.35) | 1.02 (0.85, 1.23) |
| Mixed AD/VaD | -0.72 (-1.61, 0.17) | -0.00 (-0.26, 0.26) | 1.29 (1.13, 1.47) | -0.71 (-1.61, 0.19) | 0.03 (-0.22, 0.29) | 1.28 (1.13, 1.47) |
| FTD | -2.65 (-4.16, -1.13) | 0.18 (-0.21, 0.56) | 1.03 (0.81, 1.32) | -2.78 (-4.22, -1.35) | 0.26 (-0.07, 0.59) | 0.99 (0.77, 1.27) |
| PDD | -4.64 (-6.55, -2.73) | -0.14 (-0.67, 0.40) | 1.22 (0.94, 1.59) | -4.58 (-6.53, -2.63) | -0.14 (-0.68, 0.39) | 1.18 (0.90, 1.55) |
| DLB | -4.59 (-6.36, -2.81) | 0.11 (-0.55, 0.77) | 1.68 (1.33, 2.13) | -4.52 (-6.34, -2.69) | 0.14 (-0.52, 0.80) | 1.66 (1.31, 2.11) |
| Sex | 0.53 (-0.18, 1.25) | -0.09 (-0.29, 0.11) | 0.95 (0.85, 1.05) | 0.42 (-0.31, 1.16) | -0.10 (-0.30, 0.10) | 0.95 (0.86, 1.06) |
| Association | | | | | | 0.99 (0.98, 1.01) |

1. Model 2: Adjusted for sex and age group

|  | Linear Mixed Effects Model | | Survival Model | Joint Model | | |
| --- | --- | --- | --- | --- | --- | --- |
|  | Intercept  (estimate, 95% CI) | Slope (change per year)  (estimate, 95% CI) | Survival  (HR, 95% CI) | Intercept  (estimate, 95% CI) | Slope (change per year)  (estimate, 95% CI) | Survival  (HR, 95% CI) |
| AD | Ref | Ref | Ref | Ref | Ref | Ref |
| VaD | -1.88 (-3.09, -0.67) | -0.01 (-0.34, 0.31) | 1.07 (0.89, 1.28) | -1.91 (-3.13, -0.68) | -0.02 (-0.33, 0.30) | 1.02 (0.85, 1.23) |
| Mixed AD/VaD | -1.13 (-2.01, -0.25) | -0.00 (-0.26, 0.26) | 1.20 (1.04, 1.37) | -1.18 (-2.08, -0.28) | 0.03 (-0.24, 0.29) | 1.17 (1.02, 1.34) |
| FTD | -1.48 (-3.01, 0.06) | 0.18 (-0.22, 0.58) | 1.38 (1.06, 1.78) | -1.63 (-3.15, -0.10) | 0.27 (-0.08, 0.62) | 1.31 (1.02, 1.69) |
| PDD | -4.70 (-6.59, -2.81) | -0.07 (-0.60, 0.46) | 1.34 (1.03, 1.74) | -4.64 (-6.54, -2.74) | -0.04 (-0.53, 0.46) | 1.20 (0.91, 1.58) |
| DLB | -4.58 (-6.33, -2.83) | 0.14 (-0.52, 0.79) | 1.79 (1.41, 2.26) | -4.58 (-6.33, -2.83) | 0.11 (-0.55, 0.78) | 1.65 (1.30, 2.10) |
| Sex | 0.56 (-0.15, 1.27) | -0.08 (-0.28, 0.11) | 0.96 (0.86, 1.07) | 0.61 (-0.10, 1.32) | -0.11 (-0.30, 0.09) | 0.97 (0.87, 1.08) |
| Age <65 | -3.23 (-4.36, -2.09) | -0.03 (-0.33, 0.28) | 0.45 (0.38, 0.54) | -3.24 (-4.37, -2.10) | 0.02 (-0.30, 0.33) | 0.42 (0.35, 0.51) |
| Age 65-69 | -1.01 (-2.20, 0.18) | -0.26 (-0.58, 0.07) | 0.50 (0.42, 0.60) | -1.15 (-2.36, 0.07) | -0.25 (-0.59, 0.08) | 0.49 (0.41, 0.59) |
| Age 70-74 | -0.07 (-1.11, 0.98) | -0.26 (-0.58, 0.06) | 0.70 (0.60, 0.81) | -0.06 (-1.10, 0.97) | -0.23 (-0.55, 0.09) | 0.69 (0.59, 0.80) |
| Age 75-79 | 0.13 (-0.84, 1.10) | -0.05 (-0.35, 0.24) | 0.70 (0.61, 0.81) | 0.14 (-0.86, 1.13) | -0.06 (-0.37, 0.25) | 0.70 (0.61, 0.81) |
| Age 80+ | Ref | Ref | Ref | Ref | Ref | Ref |
| Association | | | | | | - 1. (0.97, 1.00) |

1. Model 3: Adjusted for sex, age group, and MMSE

|  | Linear Mixed Effects Model | | Survival Model | Joint Model | | |
| --- | --- | --- | --- | --- | --- | --- |
|  | Intercept  (estimate, 95% CI) | Slope (change per year)  (estimate, 95% CI) | Survival  (HR, 95% CI) | Intercept  (estimate, 95% CI) | Slope (change per year)  (estimate, 95% CI) | Survival  (HR, 95% CI) |
| AD | Ref | Ref | Ref | Ref | Ref | Ref |
| VaD | -1.86 (-3.08, -0.65) | -0.03 (-0.36, 0.31) | 1.09 (0.91, 1.31) | -1.89 (-3.12, -0.68) | -0.04 (-0.35, 0.28) | 1.04 (0.87, 1.26) |
| Mixed AD/VaD | -1.12 (-2.01, -0.24) | -0.01 (-0.27, 0.26) | 1.22 (1.07, 1.39) | -1.19 (-2.09, -0.29) | 0.02 (-0.25, 0.28) | 1.19 (1.04, 1.36) |
| FTD | -1.48 (-3.02, 0.06) | 0.17 (-0.23, 0.58) | 1.45 (1.12, 1.88) | -1.68 (-3.19, -0.17) | 0.26 (-0.09, 0.62) | 1.39 (1.07, 1.80) |
| PDD | -4.67 (-6.56, -2.78) | -0.09 (-0.62, 0.46) | 1.32 (1.02, 1.72) | -4.62 (-6.52, -2.72) | -0.06 (-0.56, 0.44) | 1.16 (0.88, 1.53) |
| DLB | -4.57 (-6.32, -2.82) | 0.13 (-0.52, 0.79) | 1.81 (1.43, 2.28) | -4.58 (-6.33, -2.83) | 0.10 (-0.56, 0.77) | 1.65 (1.29, 2.11) |
| Sex | 0.55 (-0.16, 1.26) | -0.08 (-0.28, 0.12) | 0.97 (0.87, 1.08) | 0.60 (-0.11, 1.31) | -0.10 (-0.30, 0.09) | 0.98 (0.88, 1.09) |
| Age <65 | -3.21 (-4.34, -2.08) | -0.07 (-0.38, 0.24) | 0.46 (0.39, 0.56) | -3.19 (-4.32, -2.05) | -0.02 (-0.33, 0.30) | 0.43 (0.36, 0.52) |
| Age 65-69 | -0.99 (-2.18, 0.20) | -0.29 (-0.62, 0.04) | 0.52 (0.43, 0.62) | -1.11 (-2.32, 0.11) | -0.28 (-0.62, 0.06) | 0.51 (0.42, 0.61) |
| Age 70-74 | -0.06 (-1.11, 0.98) | -0.28 (-0.60, 0.04) | 0.70 (0.60, 0.82) | -0.04 (-1.07, 1.00) | -0.25 (-0.57, 0.07) | 0.69 (0.60, 0.81) |
| Age 75-79 | 0.13 (-0.84, 1.10) | -0.07 (-0.36, 0.23) | 0.71 (0.61, 0.82) | 0.14 (-0.85, 1.13) | -0.07 (-0.38, 0.24) | 0.71 (0.62, 0.82) |
| Age 80+ | Ref | Ref | Ref | Ref | Ref | Ref |
| MMSE | 0.02 (-0.05, 0.08) | 0.00 (-0.01, 0.02) | 0.97 (0.96, 0.98) | -0.01 (-0.08, 0.06) | 0.01 (-0.01, 0.02) | 0.97 (0.96, 0.98) |
| Association | | | | | | 0.98 (0.97, 1.00) |

Notes: HR, hazard ratio; CI, confidence intervals; AD, Alzheimer’s disease; VaD, vascular dementia; FTD, frontotemporal dementia; PDD, Parkinson’s disease dementia; DLB, dementia with Lewy bodies; MMSE, Mini-Mental State Examination; Ref, reference category.

Table S6. Joint model for self-rated well-being

1. Unadjusted model

|  | Linear Mixed Effects Model | | Survival Model | Joint Model | | |
| --- | --- | --- | --- | --- | --- | --- |
|  | Intercept  (estimate, 95% CI) | Slope (change per year)  (estimate, 95% CI) | Survival  (HR, 95% CI) | Intercept  (estimate, 95% CI) | Slope (change per year)  (estimate, 95% CI) | Survival  (HR, 95% CI) |
| AD | Ref | Ref | Ref | Ref | Ref | Ref |
| VaD | -4.70 (-8.85, -0.56) | -0.54 (-1.65, 0.57) | 1.02 (0.85, 1.22) | -4.64 (-9.15, -0.14) | -0.49 (-1.58, 0.61) | 0.98 (0.82, 1.18) |
| Mixed AD/VaD | -4.07 (-7.06, -1.08) | -0.24 (-1.12, 0.65) | 1.29 (1.13, 1.47) | -4.88 (-6.82, -0.93) | -0.15 (-1.03, 0.74) | 1.26 (1.10, 1.44) |
| FTD | -4.75 (-9.85, 0.36) | -0.07 (-1.44, 1.30) | 1.03 (0.81, 1.31) | -5.20 (-10.32, -0.07) | -0.13 (-1.30, 1.03) | 0.99 (0.78, 1.26) |
| PDD | -14.71 (-21.17, -8.25) | -0.66 (-2.35, 1.24) | 1.21 (0.93, 1.57) | -14.27 (-20.64, -7.90) | -0.91 (-2.72, 0.89) | 1.11 (0.85, 1.46) |
| DLB | -15.28 (-21.07, -9.48) | -0.48 (-2.55, 1.59) | 1.62 (1.29, 2.04) | -14.51 (-20.36, -8.66) | -0.68 (-2.72, 1.37) | 1.52 (1.20, 1.93) |
| Association | | | | | | 1.00 (0.99, 1.00) |

1. Model 1: Adjusted for sex

|  | Linear Mixed Effects Model | | Survival Model | Joint Model | | |
| --- | --- | --- | --- | --- | --- | --- |
|  | Intercept  (estimate, 95% CI) | Slope (change per year)  (estimate, 95% CI) | Survival  (HR, 95% CI) | Intercept  (estimate, 95% CI) | Slope (change per year)  (estimate, 95% CI) | Survival  (HR, 95% CI) |
| AD | Ref | Ref | Ref | Ref | Ref | Ref |
| VaD | -4.83 (-8.98, -0.68) | -0.56 (-1.67, 0.55) | 1.03 (0.85, 1.23) | -4.49 (-8.86, -0.12) | -0.51 (-1.60, 0.59) | 0.99 (0.82, 1.19) |
| Mixed AD/VaD | -4.13 (-7.11, -1.14) | -0.24 (-1.12, 0.65) | 1.29 (1.13, 1.47) | -4.02 (-6.97, -1.07) | -0.15 (-1.03, 0.73) | 1.26 (1.10, 1.44) |
| FTD | -4.95 (-10.06, 0.16) | -0.09 (-1.47, 1.29) | 1.04 (0.82, 1.33) | -5.15 (-10.42, 0.11) | -0.15 (-1.35, 1.05) | 1.00 (0.78, 1.27) |
| PDD | -15.27 (-21.75, -8.79) | -0.61 (-2.51, 1.29) | 1.22 (0.94, 1.58) | -14.89 (-21.35, -8.43) | -0.85 (-2.76, 1.06) | 1.12 (0.85, 1.47) |
| DLB | -15.89 (-21.71, -10.07) | -0.48 (-2.56, 1.59) | 1.65 (1.31, 2.07) | -15.15 (-21.03, -9.27) | -0.67 (-2.73, 1.39) | 1.54 (1.21, 1.96) |
| Sex | 2.33 (-0.08, 4.75) | -0.04 (-0.72, 0.64) | 0.94 (0.85, 1.05) | 2.48 (0.07, 4.89) | -0.03 (-0.73, 0.66) | 0.96 (0.86, 1.07) |
| Association | | | | | | 1.00 (0.99, 1.00) |

1. Model 2: Adjusted for sex and age group

|  | Linear Mixed Effects Model | | Survival Model | Joint Model | | |
| --- | --- | --- | --- | --- | --- | --- |
|  | Intercept  (estimate, 95% CI) | Slope (change per year)  (estimate, 95% CI) | Survival  (HR, 95% CI) | Intercept  (estimate, 95% CI) | Slope (change per year)  (estimate, 95% CI) | Survival  (HR, 95% CI) |
| AD | Ref | Ref | Ref | Ref | Ref | Ref |
| VaD | -5.19 (-9.30, -1.08) | -0.54 (-1.65, 0.57) | 1.06 (0.88, 1.27) | -5.76 (-10.29, -1.23) | -0.51 (-1.63, 0.62) | 1.01 (0.84, 1.22) |
| Mixed AD/VaD | -5.10 (-8.09, -2.10) | -0.12 (-1.01, 0.78) | 1.20 (1.05, 1.37) | -5.17 (-8.12, -2.23) | -0.09 (-0.98, 0.80) | 1.16 (1.01, 1.33) |
| FTD | -2.17 (-7.38, 3.04) | -0.40 (-1.83, 1.03) | 1.39 (1.08, 1.79) | -2.65 (-7.64, 2.35) | -0.53 (-1.88, 0.82) | 1.33 (1.03, 1.72) |
| PDD | -14.28 (-20.75, -7.82) | -0.71 (-2.61, 1.19) | 1.34 (1.03, 1.74) | -13.81 (-20.24, -7.38) | -0.94 (-2.84, 0.97) | 1.17 (0.89, 1.54) |
| DLB | -15.64 (-21.43, -9.86) | -0.38 (-2.45, 1.70) | 1.74 (1.38, 2.20) | -14.62 (-20.38, -8.86) | -0.67 (-2.71, 1.37) | 1.58 (1.24, 2.01) |
| Sex | 2.38 (-0.02, 4.78) | -0.04 (-0.73, 0.64) | 0.96 (0.86, 1.06) | 2.32 (-0.08, 4.71) | -0.15 (-0.85, 0.56) | 0.99 (0.88, 1.09) |
| Age <65 | -6.89 (-10.73, -3.04) | 0.76 (-0.30, 1.82) | 0.45 (0.37, 0.53) | -7.06 (-11.07, -3.05) | 0.83 (-0.37, 2.03) | 0.43 (0.36, 0.52) |
| Age 65-69 | -3.91 (-7.92, 0.10) | -0.11 (-1.23, 1.00) | 0.50 (0.42, 0.60) | -3.16 (-7.06, 0.74) | -0.03 (-1.14, 1.09) | 0.50 (0.41, 0.59) |
| Age 70-74 | -4.44 (-7.98, -0.90) | 0.63 (-0.47, 1.73) | 0.69 (0.59, 0.80) | -4.31 (-7.94, -0.67) | 0.59 (-0.54, 1.72) | 0.68 (0.59, 0.79) |
| Age 75-79 | 1.25 (-2.02, 4.52) | -0.27 (-1.28, 0.75) | 0.70 (0.60, 0.80) | 1.56 (-1.63, 4.75) | -0.30 (-1.29, 0.69) | 0.70 (0.61, 0.80) |
| Age 80+ | Ref | Ref | Ref | Ref | Ref | Ref |
| Association | | | | | | - 1. (0.99, 1.00) |

1. Model 3: Adjusted for sex, age group, and MMSE

|  | Linear Mixed Effects Model | | Survival Model | Joint Model | | |
| --- | --- | --- | --- | --- | --- | --- |
|  | Intercept  (estimate, 95% CI) | Slope (change per year)  (estimate, 95% CI) | Survival  (HR, 95% CI) | Intercept  (estimate, 95% CI) | Slope (change per year)  (estimate, 95% CI) | Survival  (HR, 95% CI) |
| AD | Ref | Ref | Ref | Ref | Ref | Ref |
| VaD | -5.12 (-9.23, -1.00) | -0.59 (-1.71, 0.52) | 1.08 (0.90, 1.30) | -5.64 (-10.17, -1.12) | -0.62 (-1.72, 0.49) | 1.03 (0.86, 1.24) |
| Mixed AD/VaD | -5.07 (-8.06, -2.07) | -0.11 (-1.01, 0.79) | 1.22 (1.07, 1.40) | -5.15 (-8.08, -2.22) | -0.12 (-1.00, 0.77) | 1.19 (1.03, 1.35) |
| FTD | -2.10 (-7.31, 3.12) | -0.48 (-1.92, 0.96) | 1.48 (1.14, 1.89) | -2.40 (-7.33, 2.53) | -0.70 (-1.99, 0.59) | 1.41 (1.09, 1.82) |
| PDD | -14.13 (-20.60, -7.66) | -0.80 (-2.71, 1.11) | 1.32 (1.02, 1.72) | -13.79 (-20.25, -7.33) | -0.94 (-2.93, 1.04) | 1.16 (0.88, 1.53) |
| DLB | -15.64 (-21.42, -9.86) | -0.37 (-2.45, 1.71) | 1.76 (1.40, 2.22) | -14.53 (-20.29, -8.79) | -0.72 (-2.77, 1.34) | 1.59 (1.25, 2.03) |
| Sex | 2.39 (-0.01, 4.79) | -0.08 (-0.76, 0.61) | 0.97 (0.87, 1.08) | 2.45 (0.08, 4.82) | -0.18 (-0.85, 0.50) | 0.99 (0.89, 1.10) |
| Age <65 | -6.6 (-10.71, -3.01) | 0.71 (-0.37, 1.78) | 0.46 (0.38, 0.55) | -7.32 (-11.23, -3.41) | 0.87 (-0.22, 1.96) | 0.45 (0.37, 0.54) |
| Age 65-69 | -3.85 (-7.86, 0.17) | -0.16 (-1.29, 0.96) | 0.52 (0.43, 0.62) | -2.97 (-6.87, 0.93) | -0.09 (-1.22, 1.05) | 0.52 (0.43, 0.62) |
| Age 70-74 | -4.46 (-8.00, -0.92) | 0.65 (-0.46, 1.76) | 0.69 (0.60, 0.81) | -4.28 (-7.87, -0.68) | 0.62 (-0.49, 1.71) | 0.69 (0.59, 0.80) |
| Age 75-79 | 1.23 (-2.04, 4.50) | -0.26 (-1.28, 0.76) | 0.70 (0.61, 0.81) | 1.59 (-1.61, 4.80) | -0.30 (-1.32, 0.71) | 0.71 (0.61, 0.82) |
| Age 80+ | Ref | Ref | Ref | Ref | Ref | Ref |
| MMSE | -0.02 (-0.25, 0.21) | 0.03 (-0.03, 0.08) | 0.97 (0.96, 0.98) | -0.09 (-0.33, 0.14) | 0.04 (-0.01, 0.09) | 0.97 (0.96, 0.99) |
| Association | | | | | | 0.98 (0.97, 1.00) |

Notes: HR, hazard ratio; CI, confidence intervals; AD, Alzheimer’s disease; VaD, vascular dementia; FTD, frontotemporal dementia; PDD, Parkinson’s disease dementia; DLB, dementia with Lewy bodies; MMSE, Mini-Mental State Examination; Ref, reference category.

Table S7. Joint model for informant-rated quality of life

1. Unadjusted model

|  | Linear Mixed Effects Model | | Survival Model | Joint Model | | |
| --- | --- | --- | --- | --- | --- | --- |
|  | Intercept  (estimate, 95% CI) | Slope (change per year)  (estimate, 95% CI) | Survival  (HR, 95% CI) | Intercept  (estimate, 95% CI) | Slope (change per year)  (estimate, 95% CI) | Survival  (HR, 95% CI) |
| AD | Ref | Ref | Ref | Ref | Ref | Ref |
| VaD | -2.21 (-3.52, -0.89) | 0.44 (0.07, 0.81) | 1.08 (0.88, 1.34) | -2.30 (-3.41 - -1.18) | 0.40 (0.13 – 0.68) | 1.07 (0.86, 1.32) |
| Mixed AD/VaD | -1.14 (-2.10, -0.18) | -0.09 (-0.36, 0.18) | 1.26 (1.08, 1.46) | -1.13 (-2.10 - -0.16) | -0.17 (-0.44 – 0.11) | 1.11 (0.95, 1.31) |
| FTD | -1.62 (-3.26, 0.01) | 0.15 (-0.29, 0.59) | 0.83 (0.61, 1.12) | -2.02 (-3.34 - -0.70) | 0.31 (0.05 – 0.56) | 0.77 (0.56, 1.04) |
| PDD | -2.69 (-4.57, -0.82) | -0.08 (-0.63, 0.46) | 1.21 (0.92, 1.61) | -2.86 (-4.38 - -1.34) | 0.10 (-0.44 – 0.61) | 1.05 (0.79, 1.39) |
| DLB | -2.09 (-3.90, -0.28) | -0.51 (-1.08, 0.06) | 1.45 (1.11, 1.89) | -1.88 (-3.64 - -0.12) | -0.75 (-1.32 - -0.17) | 1.15 (0.88, 1.52) |
| Association | | | | | | 0.93 (0.91, 0.94) |

1. Model 1: Adjusted for sex of person with dementia, and kin relationship

|  | Linear Mixed Effects Model | | Survival Model | Joint Model | | |
| --- | --- | --- | --- | --- | --- | --- |
|  | Intercept  (estimate, 95% CI) | Slope (change per year)  (estimate, 95% CI) | Survival  (HR, 95% CI) | Intercept  (estimate, 95% CI) | Slope (change per year)  (estimate, 95% CI) | Survival  (HR, 95% CI) |
| AD | Ref | Ref | Ref | Ref | Ref | Ref |
| VaD | -2.03 (-3.34, -0.73) | 0.43 (0.06, 0.80) | 1.07 (0.87, 1.33) | -2.28 (-3.41, -1.15) | 0.39 (0.09, 0.68) | 1.07 (0.86, 1.33) |
| Mixed AD/VaD | -0.94 (-1.89, 0.01) | -0.12 (-0.39, 0.16) | 1.21 (1.06, 1.43) | -0.84 (-1.79, 0.10) | -0.18 (-0.45, 0.08) | 1.10 (0.94, 1.28) |
| FTD | -1.83 (-3.44, -0.21) | 0.21 (-0.23, 0.64) | 0.85 (0.63, 1.16) | -2.19 (-3.52, -0.87) | 0.33 (0.06, 0.59) | 0.79 (0.58, 1.07) |
| PDD | -2.95 (-4.81, -1.09) | -0.03 (-0.59, 0.51) | 1.26 (0.95, 1.67) | -2.59 (-4.35, -0.83) | -0.31 (-0.68, 0.07) | 1.07 (0.80, 1.43) |
| DLB | -2.23 (-4.03, -0.43) | -0.46 (-1.03, 0.11) | 1.47 (1.12, 1.92) | -2.25 (-4.00, -0.49) | -0.72 (-1.29, -0.15) | 1.16 (0.88, 1.53) |
| Sex | -0.59 (-1.40, 0.21) | -0.08 (-0.31, 0.14) | 1.03 (0.90, 1.17) | -0.30 (-1.10, 0.49) | -0.03 (-0.25, 0.18) | 0.97 (0.85, 1.11) |
| Family/friend carer | -2.92 (-3.94, -1.91) | 0.30 (-0.01, 0.60) | 1.32 (1.13, 1.55) | -2.82 (-3.82, -1.82) | 0.22 (-0.05, 0.49) | 1.14 (0.97, 1.35) |
| Spouse carer | Ref | Ref | Ref | Ref | Ref | Ref |
| Association | | | | | | 0.93 (0.91, 0.94) |

1. Model 2: Adjusted for sex and age group of the person with dementia, and kin relationship

|  | Linear Mixed Effects Model | | Survival Model | Joint Model | | |
| --- | --- | --- | --- | --- | --- | --- |
|  | Intercept  (estimate, 95% CI) | Slope (change per year)  (estimate, 95% CI) | Survival  (HR, 95% CI) | Intercept  (estimate, 95% CI) | Slope (change per year)  (estimate, 95% CI) | Survival  (HR, 95% CI) |
| AD | Ref | Ref | Ref | Ref | Ref | Ref |
| VaD | -1.95 (-3.26, -0.65) | 0.39 (0.03, 0.76) | 1.11 (0.90, 1.37) | -1.96 (-3.07, -0.85) | 0.33 (0.06, 0.60) | 1.08 (0.87, 1.42) |
| Mixed AD/VaD | -0.98 (-1.94, -0.02) | -0.08 (-0.36, 0.19) | 1.17 (1.00, 1.37) | -0.95 (-1.91, 0.01) | -0.07 (-0.32, 0.18) | 1.03 (0.88, 1.19) |
| FTD | -1.66 (-3.33, 0.01) | 0.08 (-0.38, 0.53) | 1.13 (0.83, 1.55) | -1.77 (-3.15, -0.40) | 0.18 (-0.10, 0.45) | 1.02 (0.74, 1.34) |
| PDD | -3.14 (-5.01, -1.26) | 0.04 (-0.51, 0.59) | 1.30 (0.97, 1.72) | -3.34 (-5.13, -1.55) | 0.23 (-0.15, 0.60) | 1.09 (0.82, 1.52) |
| DLB | -2.26 (-4.07, -0.46) | -0.45 (-1.02, 0.11) | 1.52 (1.16, 2.00) | -2.28 (-4.09, -0.48) | -0.57 (-1.16, 0.02) | 1.20 (0.90, 1.64) |
| Sex | -0.64 (-1.45, 0.17) | -0.05 (-0.27, 0.18) | 0.99 (0.87, 1.13) | -0.16 (-0.79, 0.47) | -0.09 (-0.34, 0.15) | 0.94 (0.82, 1.08) |
| Age <65 | -0.45 (-1.72, 0.82) | 0.33 (-0.02, 0.68) | 0.45 (0.36, 0.56) | -1.09 (-2.41, 0.24) | 0.65 (0.37, 0.94) | 0.47 (0.38, 0.58) |
| Age 65-69 | -0.35 (-1.64, 0.94) | 0.47 (0.11, 0.82) | 0.52 (0.42, 0.64) | -0.48 (-1.63, 0.67) | 0.57 (0.26, 0.89) | 0.55 (0.44, 0.68) |
| Age 70-74 | 0.33 (-0.81, 1.48) | -0.08 (-0.43, 0.26) | 0.72 (0.61, 0.87) | 0.46 (-0.34, 1.26) | -0.11 (-0.44, 0.22) | 0.71 (0.59, 0.85) |
| Age 75-79 | -0.06 (-1.14, 1.03) | 0.14 (-0.19, 0.47) | 0.73 (0.61, 0.86) | 0.03 (-1.07, 1.13) | 0.31 (-0.01, 0.64) | 0.73 (0.62, 0.87) |
| Age 80+ | Ref | Ref | Ref | Ref | Ref | Ref |
| Family/friend carer | -2.98 (-4.04, -1.91) | 0.38 (0.06, 0.70) | 1.10 (0.93, 1.30) | -2.73 (-3.69, -1.77) | 0.24 (-0.01, 0.50) | 0.94 (0.79, 1.12) |
| Spouse carer | Ref | Ref | Ref | Ref | Ref | Ref |
| Association | | | | | | - 1. (0.91, 0.93) |

1. Model 3: Adjusted for sex, age group and MMSE of the person with dementia, and kin relationship

|  | Linear Mixed Effects Model | | Survival Model | Joint Model | | |
| --- | --- | --- | --- | --- | --- | --- |
|  | Intercept  (estimate, 95% CI) | Slope (change per year)  (estimate, 95% CI) | Survival  (HR, 95% CI) | Intercept  (estimate, 95% CI) | Slope (change per year)  (estimate, 95% CI) | Survival  (HR, 95% CI) |
| AD | Ref | Ref | Ref | Ref | Ref | Ref |
| VaD | -1.57 (-2.85, -0.29) | 0.15 (-0.18, 0.47) | 1.11 (0.90, 1.38) | -1.63 (-3.01, -0.26) | 0.13 (-0.19, 0.46) | 1.06 (0.85, 1.31) |
| Mixed AD/VaD | -0.71 (-1.66, 0.24) | -0.19 (-0.45, 0.07) | 1.16 (1.00, 1.36) | -0.70 (-1.65, 0.25) | -0.18 (-0.44, 0.09) | 1.09 (0.93, 1.27) |
| FTD | -1.19 (-2.84, 0.46) | 0.05 (-0.37, 0.46) | 1.16 (0.84, 1.59) | -1.22 (-2.96, 0.53) | 0.03 (-0.35, 0.41) | 1.06 (0.76, 1.46) |
| PDD | -2.48 (-4.34, -0.63) | -0.26 (-0.78, 0.26) | 1.28 (0.97, 1.70) | -2.56 (-4.36, -0.76) | -0.28 (-0.73, 0.16) | 1.11 (0.83, 1.49) |
| DLB | -2.57 (-4.36, -0.78) | -0.11 (-0.67, 0.46) | 1.51 (1.15, 1.98) | -2.31 (-4.10, -0.51) | -0.18 (-0.75, 0.38) | 1.32 (1.00, 1.75) |
| Sex | -0.68 (-1.47, 0.12) | -0.23 (-0.44, -0.02) | 1.00 (0.87, 1.14) | -0.45 (-1.26, 0.37) | -0.25 (-0.48, -0.03) | 0.92 (0.81, 1.06) |
| Age <65 | -0.91 (-2.17, 0.35) | 0.33 (0.00, 0.66) | 0.46 (0.38, 0.56) | -1.21 (-2.56, 0.13) | 0.47 (0.10, 0.84) | 0.47 (0.37, 0.58) |
| Age 65-69 | -0.80 (-2.08, 0.47) | 0.39 (0.05, 0.72) | 0.53 (0.43, 0.65) | -0.61 (-1.92, 0.70) | 0.47 (0.13, 0.82) | 0.54 (0.44, 0.67) |
| Age 70-74 | -0.15 (-1.28, 0.99) | 0.01 (-0.32, 0.34) | 0.73 (0.61, 0.87) | -0.16 (-1.27, 0.96) | 0.04 (-0.28, 0.36) | 0.73 (0.61, 0.87) |
| Age 75-79 | -0.44 (-1.51, 0.63) | 0.21 (-0.10, 0.52) | 0.74 (0.62, 0.87) | -0.57 (-1.67, 0.52) | 0.29 (-0.03, 0.62) | 0.75 (0.63, 0.90) |
| Age 80+ | Ref | Ref | Ref | Ref | Ref | Ref |
| Family/friend carer | -2.93 (-3.99, -1.88) | 0.34 (0.04, 0.64) | 1.08 (0.91, 1.28) | -2.88 (-3.94, -1.82) | 0.29 (0.01, 0.57) | 0.92 (0.84, 1.19) |
| Spouse carer | Ref | Ref | Ref | Ref | Ref | Ref |
| MMSE | 0.27 (0.21, 0.33) | -0.01 (-0.02, 0.01) | 0.98 (0.97, 1.00) | 0.23 (0.17, 0.30) | 0.00 (-0.02, 0.01) | 1.00 (0.99, 1.02) |
| Association | | | | | | 0.95 (0.93, 0.96) |

Notes: HR, hazard ratio; CI, confidence intervals; AD, Alzheimer’s disease; VaD, vascular dementia; FTD, frontotemporal dementia; PDD, Parkinson’s disease dementia; DLB, dementia with Lewy bodies; MMSE, Mini-Mental State Examination; Ref, reference category.

Table S8. Joint model for informant-rated satisfaction with life

1. Unadjusted model

|  | Linear Mixed Effects Model | | Survival Model | Joint Model | | |
| --- | --- | --- | --- | --- | --- | --- |
|  | Intercept  (estimate, 95% CI) | Slope (change per year)  (estimate, 95% CI) | Survival  (HR, 95% CI) | Intercept  (estimate, 95% CI) | Slope (change per year)  (estimate, 95% CI) | Survival  (HR, 95% CI) |
| AD | Ref | Ref | Ref | Ref | Ref | Ref |
| VaD | -2.52 (-3.99, -1.04) | 0.63 (0.23, 1.03) | 1.10 (0.89, 1.35) | -2.43 (-3.87, -0.99) | 0.73 (0.36, 1.09) | 1.07 (0.87, 1.32) |
| Mixed AD/VaD | -0.34 (-1.43, 0.76) | 0.03 (-0.28, 0.34) | 1.26 (1.08, 1.46) | -0.27 (-1.38, 0.85) | 0.02 (-0.30, 0.34) | 1.24 (1.07, 1.44) |
| FTD | -1.28 (-3.16, 0.61) | 0.01 (-0.48, 0.49) | 0.83 (0.62, 1.12) | -1.68 (-3.56, 0.20) | 0.11 (-0.27, 0.49) | 0.82 (0.60, 1.10) |
| PDD | -5.85 (-8.01, -3.68) | 0.28 (-0.33, 0.89) | 1.21 (0.91, 1.60) | -5.87 (-7.97, -3.76) | 0.32 (-0.36, 1.00) | 1.01 (0.76, 1.34) |
| DLB | -2.76 (-4.86, -0.66) | -0.66 (-1.33, 0.01) | 1.46 (1.12, 1.90) | -2.52 (-5.04, -0.21) | -0.80 (-1.48, -0.11) | 1.17 (0.88, 1.54) |
| Association | | | | | | 0.96 (0.94, 0.97) |

1. Model 1: Adjusted for sex of the person with dementia, and kin relationship

|  | Linear Mixed Effects Model | | Survival Model | Joint Model | | |
| --- | --- | --- | --- | --- | --- | --- |
|  | Intercept  (estimate, 95% CI) | Slope (change per year)  (estimate, 95% CI) | Survival  (HR, 95% CI) | Intercept  (estimate, 95% CI) | Slope (change per year)  (estimate, 95% CI) | Survival  (HR, 95% CI) |
| AD | Ref | Ref | Ref | Ref | Ref | Ref |
| VaD | -2.46 (-3.94, -0.98) | 0.61 (0.20, 1.02) | 1.09 (0.88, 1.34) | -2.40 (-3.86, -0.95) | 0.75 (0.35, 1.15) | 1.07 (0.86, 1.32) |
| Mixed AD/VaD | -0.26 (-1.34, 0.84) | -0.01 (-0.32, 0.31) | 1.23 (1.05, 1.43) | -0.12 (-1.22, 0.98) | 0.01 (-0.31, 0.32) | 1.21 (1.04, 1.41) |
| FTD | -1.39 (-3.27, 0.50) | 0.09 (-0.39, 0.57) | 0.86 (0.64, 1.16) | -1.75 (-3.62, 0.12) | 0.17 (-0.22, 0.56) | 0.85 (0.63, 1.14) |
| PDD | -6.00 (-8.18, -3.83) | 0.38 (-0.24, 1.01) | 1.26 (0.95, 1.67) | -5.86 (-10.16, -1.56) | 0.28 (-2.54, 3.11) | 1.05 (0.79, 1.41) |
| DLB | -2.87 (-4.98, -0.75) | -0.61 (-1.31, 0.08) | 1.48 (1.13, 1.94) | -2.74 (-5.14, -0.34) | -0.75 (-1.44, -0.06) | 1.19 (0.89, 1.58) |
| Sex | 0.06 (-0.87, 0.99) | -0.15 (-0.41, 0.10) | 1.02 (0.89, 1.16) | 0.01 (-0.92, 0.94) | -0.17 (-0.46, 0.11) | 1.00 (0.87, 1.14) |
| Family/friend carer | -0.93 (-2.09, 0.23) | 0.20 (-0.15, 0.55) | 1.33 (1.14, 1.56) | -1.01 (-2.16, 0.15) | 0.12 (-0.24, 0.48) | 1.31 (1.11, 1.54) |
| Spouse carer | Ref | Ref | Ref | Ref | Ref | Ref |
| Association | | | | | | - 1. (0.94, 0.97) |

1. Model 2: Adjusted for sex and age group of the person with dementia, and kin relationship

|  | Linear Mixed Effects Model | | Survival Model | Joint Model | | |
| --- | --- | --- | --- | --- | --- | --- |
|  | Intercept  (estimate, 95% CI) | Slope (change per year)  (estimate, 95% CI) | Survival  (HR, 95% CI) | Intercept  (estimate, 95% CI) | Slope (change per year)  (estimate, 95% CI) | Survival  (HR, 95% CI) |
| AD | Ref | Ref | Ref | Ref | Ref | Ref |
| VaD | -2.58 (-4.05, -1.11) | 0.63 (0.23, 1.03) | 1.12 (0.91, 1.38) | -2.57 (-4.03, -1.12) | 0.73 (0.34, 1.11) | 1.10 (0.89, 1.36) |
| Mixed AD/VaD | -0.54 (-1.64, 0.56) | 0.03 (-0.29, 0.34) | 1.17 (1.00, 1.36) | -0.47 (-1.56, 0.62) | 0.06 (-0.25, 0.38) | 1.14 (0.98, 1.33) |
| FTD | -0.65 (-2.58, 1.29) | 0.02 (-0.49, 0.53) | 1.14 (0.83, 1.55) | -0.81 (-2.74, 1.13) | 0.04 (-0.39, 0.47) | 1.16 (0.85, 1.58) |
| PDD | -6.02 (-8.20, -3.84) | 0.39 (-0.22, 1.01) | 1.29 (0.97, 1.72) | -5.91 (-8.00, -3.81) | 0.43 (-0.16, 1.02) | 1.06 (0.79, 1.41) |
| DLB | -2.83 (-4.94, -0.72) | -0.59 (-1.26, 0.09) | 1.53 (1.17, 2.01) | -2.54 (-4.80, -0.28) | -0.75 (-1.43, -0.07) | 1.19 (0.89, 1.58) |
| Sex | -0.11 (-1.04, 0.82) | -0.13 (-0.38, 0.13) | 0.98 (0.86, 1.12) | -0.23 (-1.17, 0.70) | -0.11 (-0.35, 0.14) | 0.97 (0.84, 1.10) |
| Age <65 | -2.74 (-4.22, -1.27) | 0.19 (-0.21, 0.58) | 0.45 (0.36, 0.56) | -2.97 (-4.47, -1.47) | 0.39 (-0.01, 0.79) | 0.40 (0.32, 0.49) |
| Age 65-69 | -0.90 (-2.37, 0.58) | 0.04 (-0.36, 0.44) | 0.52 (0.43, 0.65) | -1.11 (-2.72, 0.50) | 0.17 (-0.24, 0.57) | 0.50 (0.40, 0.61) |
| Age 70-74 | -1.44 (-2.76, -0.13) | -0.02 (-0.41, 0.37) | 0.74 (0.62, 0.88) | -1.62 (-2.94, -0.30) | 0.03 (-0.35, 0.42) | 0.69 (0.57, 0.82) |
| Age 75-79 | -0.91 (-2.16, 0.33) | 0.21 (-0.16, 0.59) | 0.73 (0.62, 0.86) | -0.95 (-2.20, 0.29) | 0.24 (-0.13, 0.61) | 0.72 (0.60, 0.85) |
| Age 80+ | Ref | Ref | Ref | Ref | Ref | Ref |
| Family/friend carer | -1.51 (-2.72, -0.30) | 0.22 (-0.13, 0.57) | 1.12 (0.95, 1.32) | -1.74 (-2.97, -0.51) | 0.20 (-0.16, 0.55) | 1.08 (0.91, 1.28) |
| Spouse carer | Ref | Ref | Ref | Ref | Ref | Ref |
| Association | | | | | | - 1. (0.94, 0.96) |

1. Model 3: Adjusted for sex, age group and MMSE of the person with dementia, and kin relationship

|  | Linear Mixed Effects Model | | Survival Model | Joint Model | | |
| --- | --- | --- | --- | --- | --- | --- |
|  | Intercept  (estimate, 95% CI) | Slope (change per year)  (estimate, 95% CI) | Survival  (HR, 95% CI) | Intercept  (estimate, 95% CI) | Slope (change per year)  (estimate, 95% CI) | Survival  (HR, 95% CI) |
| AD | Ref | Ref | Ref | Ref | Ref | Ref |
| VaD | -2.27 (-3.74, -0.81) | 0.40 (0.01, 0.80) | 1.13 (0.92, 1.40) | -2.28 (-3.72, -0.83) | 0.41 (0.03, 0.79) | 1.09 (0.88, 1.34) |
| Mixed AD/VaD | -0.46 (-1.60, 0.65) | -0.01 (-0.35, 0.31) | 1.17 (1.00, 1.36) | -0.46 (-1.55, 0.63) | -0.02 (-0.35, 0.31) | 1.15 (0.98, 1.34) |
| FTD | -0.30 (-2.24, 1.64) | 0.00 (-0.52, 0.53) | 1.15 (0.83, 1.57) | -0.39 (-2.33, 1.55) | -0.01 (-0.59, 0.56) | 1.15 (0.84, 1.57) |
| PDD | -5.73 (-7.92, -3.55) | 0.23 (-0.41, 0.86) | 1.28 (0.96, 1.70) | -5.71 (-7.85, -3.58) | 0.16 (-0.43, 0.76) | 1.08 (0.81, 1.45) |
| DLB | -2.76 (-4.89, -0.63) | -0.56 (-1.28, 0.16) | 1.52 (1.16, 1.99) | -2.49 (-4.68, -0.29) | -0.74 (-1.49, 0.02) | 1.26 (0.94, 1.67) |
| Sex | -0.19 (-1.12, 0.74) | -0.21 (-0.47, 0.05) | 0.99 (0.87, 1.13) | -0.08 (-1.01, 0.85) | -0.23 (-0.48, 0.03) | 0.97 (0.85, 1.11) |
| Age <65 | -3.42 (-4.90, -1.94) | 0.36 (-0.04, 0.77) | 0.46 (0.37, 0.57) | -3.65 (-5.15, -2.16) | 0.42 (0.02, 0.83) | 0.42 (0.34, 0.42) |
| Age 65-69 | -1.39 (-2.87, 0.10) | 0.13 (-0.29, 0.54) | 0.54 (0.44, 0.66) | -1.25 (-2.75, 0.24) | 0.23 (-0.19, 0.65) | 0.52 (0.42, 0.64) |
| Age 70-74 | -2.08 (-3.41, -0.76) | 0.23 (-0.19, 0.64) | 0.74 (0.62, 0.89) | -2.24 (-3.57, -0.92) | 0.26 (-0.17, 0.69) | 0.72 (0.60, 0.86) |
| Age 75-79 | -1.49 (-2.74, -0.24) | 0.44 (0.06, 0.83) | 0.74 (0.63, 0.88) | -1.49 (-2.70, -0.28) | 0.49 (0.10, 0.87) | 0.75 (0.63, 0.89) |
| Age 80+ | Ref | Ref | Ref | Ref | Ref | Ref |
| Family/friend carer | -1.80 (-3.02, -0.58) | 0.43 (0.06, 0.80) | 1.11 (0.94, 1.31) | -1.83 (-3.05, -0.61) | 0.42 (0.05, 0.79) | 1.12 (0.94, 1.32) |
| Spouse carer | Ref | Ref | Ref | Ref | Ref | Ref |
| MMSE | 0.15 (0.07, 0.23) | 0.01 (-0.01, 0.02) | 0.99 (0.97, 1.00) | 0.12 (0.04, 0.19) | 0.01 (-0.01, 0.03) | 0.99 (0.98, 1.01) |
| Association | | | | | | 0.96 (0.94, 0.98) |

Notes: HR, hazard ratio; CI, confidence intervals; AD, Alzheimer’s disease; VaD, vascular dementia; FTD, frontotemporal dementia; PDD, Parkinson’s disease dementia; DLB, dementia with Lewy bodies; MMSE, Mini-Mental State Examination; Ref, reference category.

Table S9. Joint model for informant-rated well-being

1. Unadjusted model

|  | Linear Mixed Effects Model | | Survival Model | Joint Model | | |
| --- | --- | --- | --- | --- | --- | --- |
|  | Intercept  (estimate, 95% CI) | Slope (change per year)  (estimate, 95% CI) | Survival  (HR, 95% CI) | Intercept  (estimate, 95% CI) | Slope (change per year)  (estimate, 95% CI) | Survival  (HR, 95% CI) |
| AD | Ref | Ref | Ref | Ref | Ref | Ref |
| VaD | -7.42 (-11.99, -2.86) | 1.00 (-0.20, 2.19) | 1.08 (0.88, 1.34) | -8.69 (-13.25, -4.13) | 1.01 (-0.06, 2.09) | 1.01 (0.82, 1.25) |
| Mixed AD/VaD | -5.26 (-8.61, -1.92) | 0.02 (-0.90, 0.95) | 1.27 (1.09, 1.48) | -5.32 (-8.67, -1.98) | -0.04 (-0.95, 0.86) | 1.13 (0.97, 1.32) |
| FTD | -4.66 (-10.35, 1.03) | 1.61 (0.17, 3.04) | 0.85 (0.63, 1.14) | -5.95 (-11.88, -0.01) | 2.00 (0.19, 3.81) | 0.83 (0.61, 1.12) |
| PDD | -13.59 (-20.21, -6.97) | 0.35 (-1.47, 2.18) | 1.21 (0.92, 1.60) | -12.29 (-18.82, -5.77) | -0.05 (-1.59, 1.50) | 0.95 (0.71, 1.27) |
| DLB | -10.89 (-17.26, -4.54) | -1.17 (-3.18, 0.84) | 1.48 (1.14, 1.93) | -9.92 (-16.36, -3.48) | -1.76 (-3.71, 0.19) | 1.14 (0.87, 1.50) |
| Association | | | | | | - 1. (0.97, 0.98) |

1. Model 1: Adjusted for sex of the person with dementia

|  | Linear Mixed Effects Model | | Survival Model | Joint Model | | |
| --- | --- | --- | --- | --- | --- | --- |
|  | Intercept  (estimate, 95% CI) | Slope (change per year)  (estimate, 95% CI) | Survival  (HR, 95% CI) | Intercept  (estimate, 95% CI) | Slope (change per year)  (estimate, 95% CI) | Survival  (HR, 95% CI) |
| AD | Ref | Ref | Ref | Ref | Ref | Ref |
| VaD | -6.71 (-11.22, -2.20) | 0.95 (-0.24, 2.15) | 1.01 (0.87, 1.33) | -8.08 (-12.87, -3.30) | 1.16 (0.09, 2.23) | 1.02 (0.82, 1.26) |
| Mixed AD/VaD | -4.53 (-7.84, -1.22) | -0.09 (-1.01, 0.83) | 1.07 (1.06, 1.44) | -4.37 (-7.64, -1.09) | -0.14 (-1.07, 0.79) | 1.12 (0.95, 1.31) |
| FTD | -5.58 (-11.20, 0.04) | 1.87 (0.44, 3.30) | 1.24 (0.65, 1.18) | -5.74 (-11.49, 0.00) | 2.03 (0.69, 3.36) | 0.85 (0.63, 1.15) |
| PDD | -14.77 (-21.33, -8.21) | 0.61 (-1.21, 2.43) | 0.88 (0.95, 1.67) | -13.31 (-19.35, -7.27) | 0.39 (-1.11, 1.89) | 0.98 (0.73, 1.32) |
| DLB | -11.59 (-17.90, -5.27) | -0.88 (-2.89, 1.13) | 1.26 (1.16, 1.96) | -10.46 (-16.77, -4.15) | -1.38 (-3.34, 0.58) | 1.17 (0.88, 1.54) |
| Sex | -1.22 (-4.03, 1.59) | -0.58 (-1.33, 0.17) | 1.01 (0.89, 1.15) | -1.11 (-3.94, 1.72) | -0.78 (-1.54, -0.03) | 0.95 (0.83, 1.08) |
| Family/friend carer | -10.21 (-13.72, -6.70) | 1.15 (0.15, 2.16) | 1.32 (1.12, 1.54) | -10.19 (-13.60, -6.78) | 0.80 (-0.14, 1.74) | 1.14 (0.97, 1.35) |
| Spouse carer | Ref | Ref | Ref | Ref | Ref | Ref |
| Association | | | | | | - 1. (0.97, 0.98) |

1. Model 2: Adjusted for sex and age group of the person with dementia

|  | Linear Mixed Effects Model | | Survival Model | Joint Model | | |
| --- | --- | --- | --- | --- | --- | --- |
|  | Intercept  (estimate, 95% CI) | Slope (change per year)  (estimate, 95% CI) | Survival  (HR, 95% CI) | Intercept  (estimate, 95% CI) | Slope (change per year)  (estimate, 95% CI) | Survival  (HR, 95% CI) |
| AD | Ref | Ref | Ref | Ref | Ref | Ref |
| VaD | -6.52 (-11.03, -2.01) | 0.89 (-0.31, 2.08) | 1.11 (0.90, 1.37) | -7.85 (-12.26, -3.43) | 0.86 (-0.11, 1.83) | 1.04 (0.84, 1.28) |
| Mixed AD/VaD | -4.23 (-7.58, -0.88) | -0.08 (-1.01, 0.86) | 1.19 (1.02, 1.38) | -3.67 (-2.89 -0.44) | -0.12 (-0.99, 0.74) | 1.07 (0.91, 1.25) |
| FTD | -6.13 (-11.93, -0.33) | 1.71 (0.22, 3.20) | 1.17 (0.86, 1.59) | -7.06 (-13.00, -1.13) | 1.58 (0.38, 2.80) | 1.12 (0.82, 1.53) |
| PDD | -14.95 (-21.56, -8.34) | 0.71 (-1.12, 2.55) | 1.29 (0.97, 1.72) | -13.85 (-19.95, -6.20) | 0.51 (-0.76, 1.78) | 1.01 (0.77, 1.34) |
| DLB | -11.63 (-17.96, -5.30) | -0.91 (-2.91, 1.10) | 1.57 (1.20, 2.05) | -10.84 (-17.17, -4.51) | -1.51 (-3.48, 0.45) | 1.22 (0.92, 1.60) |
| Sex | -1.05 (-3.88, 1.78) | -0.56 (-1.31, 0.19) | 0.97 (0.85, 1.11) | -0.84 (-3.52, 1.84) | -0.68 (-1.27, -0.06) | 0.92 (0.80, 1.05) |
| Age <65 | 2.46 (-2.02, 6.93) | 0.31 (-0.87, 1.48) | 0.43 (0.35, 0.54) | 2.85 (-1.44, 7.13) | 0.94 (0.12, 1.76) | 0.46 (0.37, 0.58) |
| Age 65-69 | 2.95 (-1.55, 7.45) | 0.61 (-0.58, 1.81) | 0.51 (0.41, 0.62) | 2.76 (-1.52, 7.03) | 1.08 (-0.04, 2.21) | 0.56 (0.45, 0.69) |
| Age 70-74 | 1.34 (-2.64, 5.31) | -0.17 (-1.33, 1.00) | 0.72 (0.60, 0.86) | 1.94 (-1.69, 5.57) | -0.04 (-1.03, 0.95) | 0.72 (0.60, 0.87) |
| Age 75-79 | 2.17 (-1.60, 5.94) | 0.01 (-1.09, 1.11) | 0.71 (0.60, 0.85) | 2.80 (-0.93, 6.54) | 0.32 (-0.69, 1.33) | 0.75 (0.63, 0.90) |
| Age 80+ | Ref | Ref | Ref | Ref | Ref | Ref |
| Family/friend carer | -9.40 (-13.09, -5.70) | 1.24 (0.19, 2.29) | 1.09 (0.92, 1.29) | -9.52 (-12.96, -6.09) | 1.14 (0.19, 2.10) | 0.96 (0.80, 1.14) |
| Spouse carer | Ref | Ref | Ref | Ref | Ref | Ref |
| Association | | | | | | - 1. (0.97, 0.98) |

1. Model 3: Adjusted for sex, age group and MMSE of the person with dementia, and kin relationship

|  | Linear Mixed Effects Model | | Survival Model | Joint Model | | |
| --- | --- | --- | --- | --- | --- | --- |
|  | Intercept  (estimate, 95% CI) | Slope (change per year)  (estimate, 95% CI) | Survival  (HR, 95% CI) | Intercept  (estimate, 95% CI) | Slope (change per year)  (estimate, 95% CI) | Survival  (HR, 95% CI) |
| AD | Ref | Ref | Ref | Ref | Ref | Ref |
| VaD | -4.83 (-9.25, -0.41) | -0.14 (-1.30, 1.08) | 1.12 (0.91, 1.38) | -5.52 (-9.98, -1.05) | -0.24 (-1.31, 0.82) | 1.03 (0.83, 1.28) |
| Mixed AD/VaD | -3.43 (-6.73, -0.12) | -0.37 (-1.34, 0.59) | 1.18 (1.02, 1.38) | -2.87 (-6.16 0.41) | -0.34 (-1.31, 0.63) | 1.10 (0.94, 1.25) |
| FTD | -4.09 (-9.80, 1.63) | 0.59 (-0.95, 2.13) | 1.18 (0.87, 1.61) | -4.41 (-9.89, 1.07) | 0.51 (-0.78, 1.81) | 1.11 (0.81, 1.53) |
| PDD | -12.83 (-19.38, -6.27) | -0.31 (-2.21, 1.58) | 1.28 (0.97, 1.70) | -12.18 (-18.58, -5.77) | -0.42 (-2.31, 1.47) | 1.04 (0.77, 1.34) |
| DLB | -12.16 (-18.39, -5.92) | -0.01 (-2.09, 2.06) | 1.55 (1.19, 2.03) | -11.60 (-17.76, -5.44) | -0.46 (-2.46, 1.55) | 1.32 (1.00, 1.60) |
| Sex | -1.41 (-4.20, 1.38) | -0.74 (-1.51, 0.03) | 0.98 (0.86, 1.12) | -1.16 (-3.89, 1.57) | -0.92 (-1.63, -0.21) | 0.93 (0.81, 1.07) |
| Age <65 | 7.24 (-3.70, 5.15) | 0.78 (-0.43, 1.99) | 0.44 (0.36, 0.55) | 0.49 (-3.87, 4.86) | 1.04 (-0.10, 2.17) | 0.46 (0.37, 0.58) |
| Age 65-69 | 2.37 (-2.07, 6.81) | 0.37 (-0.86, 1.60) | 0.52 (0.42, 0.64) | 2.46 (-2.04, 6.96) | 0.57 (-0.64, 1.78) | 0.56 (0.45, 0.69) |
| Age 70-74 | 0.59 (-3.34, 4.53) | -0.12 (-1.32, 1.08) | 0.72 (0.61, 0.87) | 0.56 (-3.30, 4.42) | -0.12 (-1.18, 1.04) | 0.73 (0.61, 0.87) |
| Age 75-79 | 1.49 (-2.23, 5.22) | 0.18 (-0.995, 1.32) | 0.72 (0.61, 0.86) | 1.84 (-1.85, 5.54) | 0.42 (-0.67, 1.51) | 0.76 (0.64, 0.90) |
| Age 80+ | Ref | Ref | Ref | Ref | Ref | Ref |
| Family/friend carer | -8.96 (-12.62, -5.31) | 0.98 (-0.11, 2.07) | 1.08 (0.91, 1.27) | -9.52 (-13.10, -5.94) | 0.77 (-0.22, 1.76) | 0.98 (0.82, 1.17) |
| Spouse carer | Ref | Ref | Ref | Ref | Ref | Ref |
| MMSE | 0.48 (0.25, 0.71) | 0.00 (-0.05, 0.05) | 0.98 (0.97, 1.00) | 0.38 (0.15, 0.60) | 0.01 (-0.04, 0.07) | 0.99 (0.97, 1.01) |
| Association | | | | | | 0.97 (0.95, 0.99) |

Notes: HR, hazard ratio; CI, confidence intervals; AD, Alzheimer’s disease; VaD, vascular dementia; FTD, frontotemporal dementia; PDD, Parkinson’s disease dementia; DLB, dementia with Lewy bodies; MMSE, Mini-Mental State Examination; Ref, reference category.

Fig. S1. Trajectories of quality of life by diagnostic group

1. Self-rated quality of life B) Informant-rated quality of life


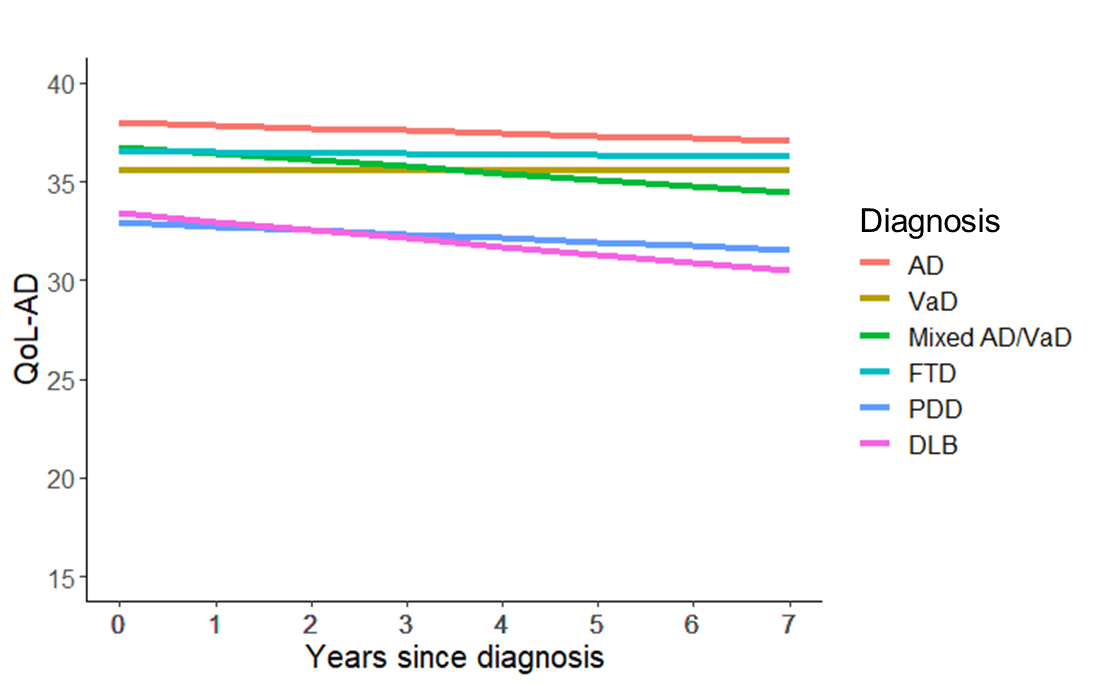

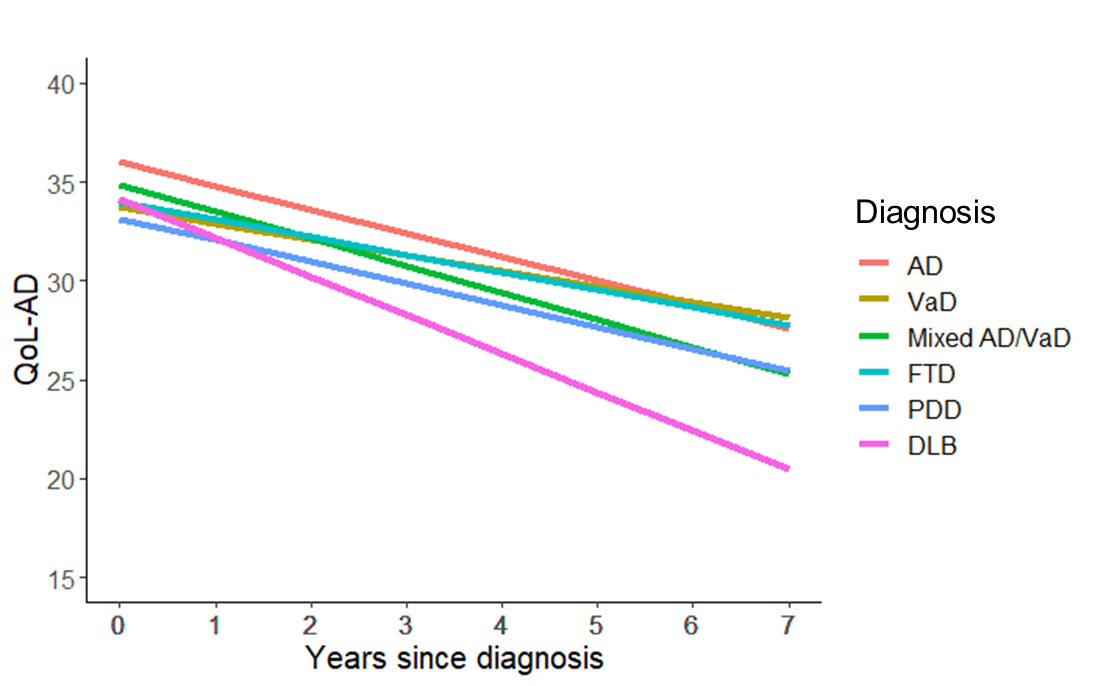


Notes: Figures are visualisations of the unadjusted model results presented in Table 2 (self-rated) and Table 3 (informant-rated) and assume a linear trend.
